# Vaccination of Gerbils with *Bm*-103 and *Bm*-RAL-2 Concurrently or as a Fusion Protein Confers Consistent and Improved Protection against *Brugia malayi* Infection

**DOI:** 10.1371/journal.pntd.0004586

**Published:** 2016-04-05

**Authors:** Sridhar Arumugam, Junfei Wei, Zhuyun Liu, David Abraham, Aaron Bell, Maria Elena Bottazzi, Peter J. Hotez, Bin Zhan, Sara Lustigman, Thomas R. Klei

**Affiliations:** 1 Department of Pathobiological Sciences, LSU School of Veterinary Medicine, Louisiana State University, Baton Rouge, Louisiana, United States of America; 2 Sabin Vaccine Institute and Texas Children’s Hospital Center for Vaccine Development, National School of Tropical Medicine, Houston, Texas, United States of America; 3 Department of Microbiology and Immunology, Sidney Kimmel Medical College at Thomas Jefferson University, Philadelphia, Pennsylvania, United States of America; 4 Laboratory of Molecular Parasitology, Lindsley F. Kimball Research Institute, New York Blood Center, New York, New York, United States of America; Uniformed Services University of the Health Sciences, UNITED STATES

## Abstract

**Background:**

The *Brugia malayi Bm*-103 and *Bm*-RAL-2 proteins are orthologous to *Onchocerca volvulus Ov*-103 and *Ov*-RAL-2, and which were selected as the best candidates for the development of an *O*. *volvulus* vaccine. The *B*. *malayi* gerbil model was used to confirm the efficacy of these *Ov* vaccine candidates on adult worms and to determine whether their combination is more efficacious.

**Methodology and Principle Findings:**

Vaccine efficacy of recombinant *Bm*-103 and *Bm*-RAL-2 administered individually, concurrently or as a fusion protein were tested in gerbils using alum as adjuvant. Vaccination with *Bm*-103 resulted in worm reductions of 39%, 34% and 22% on 42, 120 and 150 days post infection (dpi), respectively, and vaccination with *Bm*-RAL-2 resulted in worm reductions of 42%, 22% and 46% on 42, 120 and 150 dpi, respectively. Vaccination with a fusion protein comprised of *Bm*-103 and *Bm*-RAL-2 resulted in improved efficacy with significant reduction of worm burden of 51% and 49% at 90 dpi, as did the concurrent vaccination with *Bm*-103 and *Bm*-RAL-2, with worm reduction of 61% and 56% at 90 dpi. Vaccination with *Bm*-103 and *Bm*-RAL-2 as a fusion protein or concurrently not only induced a significant worm reduction of 61% and 42%, respectively, at 150 dpi, but also significantly reduced the fecundity of female worms as determined by embryograms. Elevated levels of antigen-specific IgG were observed in all vaccinated gerbils. Serum from gerbils vaccinated with *Bm*-103 and *Bm*-RAL-2 individually, concurrently or as a fusion protein killed third stage larvae *in vitro* when combined with peritoneal exudate cells.

**Conclusion:**

Although vaccination with *Bm*-103 and *Bm*-RAL-2 individually conferred protection against *B*. *malayi* infection in gerbils, a more consistent and enhanced protection was induced by vaccination with *Bm*-103 and *Bm-*RAL-2 fusion protein and when they were used concurrently. Further characterization and optimization of these filarial vaccines are warranted.

## Author Summary

Onchocerciasis and Lymphatic filariasis (LF) are debilitating neglected tropical diseases (NTDs). Practical challenges in implementation of mass drug administration (MDA) such as prolonged treatment regime requirements and the possible emergence of drug resistance will likely impede the elimination of these NTDs. Hence, the availability of an efficacious prophylactic vaccine would be an invaluable tool. The objective of the present studies was to use the *B*. *malayi*-gerbil model of filariasis as a surrogate system to test the efficacy of filarial molecules as vaccine targets for an onchocerciasis vaccine. The vaccine efficacy of *Onchocerca volvulus* recombinant proteins *Ov*-RAL-2 and *Ov*-103 was recently demonstrated using a mouse diffusion chamber model. In this communication, we provide encouraging results on the vaccine efficacy of *Bm*-RAL-2 and *Bm*-103, individually or in combination. Our data demonstrate that vaccination with *Bm*-RAL-2 and *Bm*-103 concurrently and as a fusion protein confers not only a consistent and significant protection against *B*. *malayi* infection in gerbils, but also reduces the fecundity of female worms as demonstrated in embryogram analyses. Our results support the contention that *Ov*-RAL-2 and *Ov*-103 are excellent onchocerciasis vaccine candidates and that further investigations leading to their development as a vaccine are warranted.

## Introduction

Onchocerciasis is an important neglected tropical disease (NTD) caused by the filarial parasite, *Onchocerca volvulus*. The parasite is transmitted by black fly species of the genus *Simulium* and is found in some regions of South and Central America but most commonly now in sub-Saharan areas of Africa. The Global Burden of Disease Study 2013 estimates that 16.96 million people are infected with *O*. *volvulus* [1]. It is a leading cause of blindness in Africa and produces severe skin disease and generalized morbidity in some individuals. Annual MDA programs using ivermectin to eliminate microfilaremia and reduce transmission have been effective in some areas and the elimination of *O*. *volvulus* has been targeted for 2025 by the African Programme for Onchocerciasis Control (APOC) [2]. Such activities have reduced the global prevalence of onchocerciasis by 31.2% over the last two decades [1]. Nonetheless, practical challenges have arisen that will likely impede the successful elimination of onchocerciasis in the planned time line. These include the need for repeated ivermectin treatment regime for many years [3], and the possible emergence in some endemic areas of resistance to ivermectin [4–8]. In addition, MDA cannot be implemented in areas where onchocerciasis and loiasis are co-endemic because ivermectin treatment of patients with high *Loa loa* microfilaremia may lead to severe adverse side effects including encephalopathy [9]. According to a new APOC estimate, elimination would require an estimated 1.30 billion treatments, lasting until 2045 [10]. However, a recent survey of almost 400 NTD experts indicated that many believe onchocerciasis will never be eliminated through MDA with ivermectin solely [11]. Hence, to strengthen the current elimination programs additional tools are necessary to prevent infection, cure infection and interrupt transmission, and thus complement current MDA programs [12–14]. An efficacious vaccine against onchocerciasis will be an invaluable tool in the effort to eliminate onchocerciasis from humans.

Immunity to filarial infections has been well documented in animal models for onchocerciasis and lymphatic filariasis (LF). For onchocerciasis, the mouse diffusion chamber model serves as an efficient method for identifying candidate vaccine antigens and studying protective immunity against the early stages of infection with the third-stage larvae (L3) of *O*. *volvulus*. Lange et al., 1993 reported a significant reduction in the survival of challenge parasites when mice are vaccinated with irradiated *O*. *volvulus* L3s [15]. Similarly, it was observed in cattle in natural infection settings where irradiated L3s induced protective immunity to *O*. *ochengi*, a closely related parasite [16]. In LF animal models, vaccination with irradiated L3s has also been demonstrated to be effective in permissive hosts of *Brugia* species such as cats, dogs, and Mongolian gerbils [17]. Many investigators have also shown some protective vaccine efficacy with a wide range of native and recombinant proteins of *O*. *volvulus* and *B*. *malayi* (as reviewed in [17]).

The absence of a permissive small animal model of onchocerciasis, which allows for the complete development of *O*. *volvulus* parasites, prompted us to utilize at least two complementary animal models which provide distinct advantages for validating vaccine candidates before moving forward to clinical development. This strategy is discussed in detail elsewhere [4, 14, 18]. The *O*. *volvulus–*mouse diffusion chamber model allows testing vaccines only against the L3-L4 stages of the parasite and the in depth study of the murine effector mechanisms within the confined environment of the diffusion chamber. While the *B*. *malayi*-Mongolian gerbil model lacks the availability of immunological reagents and genetically defined animals when compared with the mouse system, it does allow for the testing of vaccines in a completely permissive host, which allows for examining efficacy against all life cycle stages of the parasite including effects on the normal development of embryogenesis. Together, these two models provide a rigorous testing of homologous protein vaccines. The objective of the studies reported in the current paper is to show that the *B*. *malayi*–gerbil model of lymphatic filariasis validates the positioning of the two selected recombinant *O*. *volvulus* vaccine proteins, *Ov*-103 and *Ov*-RAL-2 [19], for a prophylactic anti-*Onchocerca* vaccine. *Ov*-RAL-2 was identified by screening an adult stage cDNA library with anti-L3 rabbit serum [20, 21], and *Ov*-103 was identified by screening an adult stage cDNA library with antiserum from *O*. *volvulus* infected chimpanzee [22].

In the current study, we tested the efficacy of *Bm*-103 and *Bm*-RAL-2, the homologs of the proven vaccine candidates of *O*. *volvulus*, *Ov*-103 and *Ov*-RAL-2, in the Mongolian gerbil model either individually, as a fusion protein or as concurrent vaccines with separate injections. *Bm*-103 and *Bm*-RAL-2 induced protection against *B*. *malayi* infection in Mongolian gerbils. Higher levels of protection were achieved when gerbils were vaccinated with *Bm*-103 and *Bm*-RAL-2 concurrently or as a fusion protein. Vaccination with *Bm*-RAL-2, *Bm*-103 and *Bm*-RAL-2 concurrently or as a fusion protein also reduced the fecundity of female worms recovered at 150 dpi as demonstrated by embryograms. Using the *B*. *malayi* infection model, we have confirmed the effectiveness of the *O*. *volvulus* orthologous antigens from *B*. *malayi*, *Bm*-103 and *Bm*-RAL-2, as efficacious vaccines also against infection with *B*. *malayi* L3s. Moreover, the present studies have also established that *Bm*-103 and *Bm*-RAL-2 could also be considered as validated vaccines to prevent LF.

## Materials and Methods

### Expression and purification of *B*. *malayi* vaccine antigens

#### *Bm*-103

*Bm*-103 (GenBank accession # XP_001891826) is a homolog of *Ov*-103 in *Brugia malayi* with 68% amino acid sequence identity. The amino acid sequence comparison between *Bm*-103 and *Ov*-103 and the phylogenetic analysis of *Bm*-103 with other nematode homologs are shown as supplementary figures (Fig S1 A–B in S1 Dataset). Even though there is no specific functional domain found in *Bm*-103 through Pfam database search, in previous studies the *O*. *volvulus* homolog *Ov*-103 was initially described as a microfilariae surface associated antigen [22]. In later studies, it was found to be also expressed by *O*. *volvulus* L3s [23], and vaccination with recombinant *Ov*-103 induced strong protective immunity in *O*. *volvulus* mouse chamber model [19]. Recent immunoelectron microscopy studies were used to localize *Bm*-103 and *Ov*-103 in *B*. *malayi* and *O*. *volvulus*, respectively, using monospecific human anti-*Ov*-103 antibodies [24] that cross reacted with the *Bm*-103 protein. In *O*. *volvulus*, *Ov-*103 was confirmed to be localized in the hypodermis and cuticle of adult female worms, and on the surface of MF [22], but it also now newly shown that it is localized on the surface and the glandular esophagus of L3s (Fig S2 in S1 Dataset). Similarly, *Bm*-103 was found to be localized in the hypodermis and cuticle of adult female worm, L3 and MF (Fig S2 in S1 Dataset).

**Fig 1:**
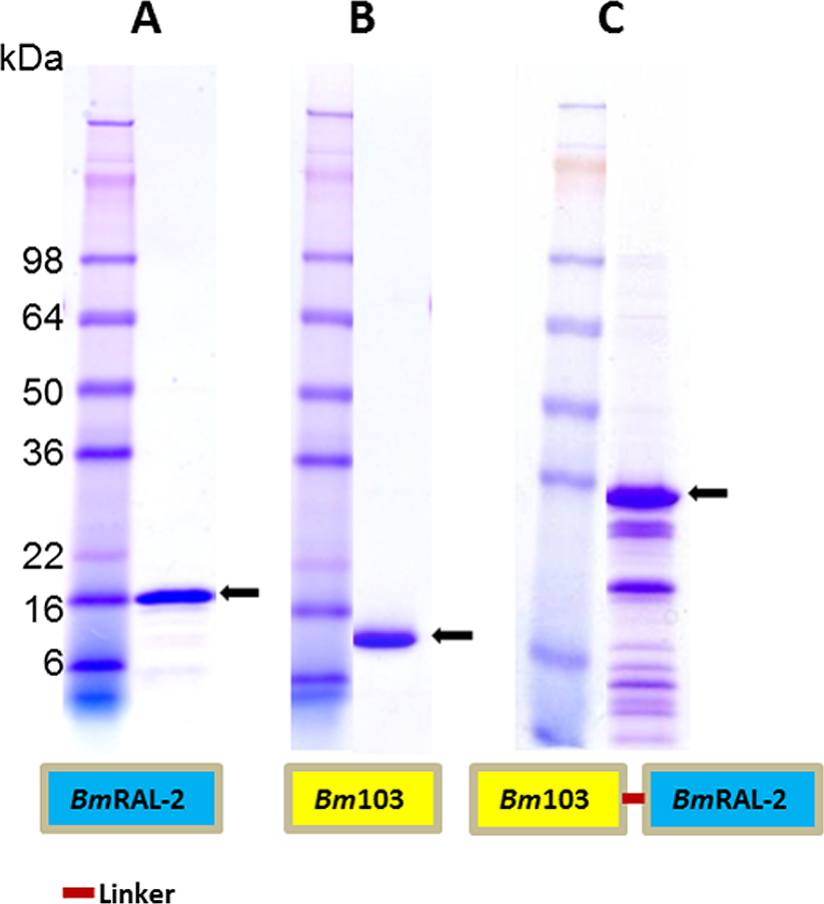
SDS-PAGE of purified *Brugia malayi* recombinant proteins, *Bm*-RAL-2, *Bm*-103 and *Bm*-RAL-2—*Bm*-103 fusion protein. Two μg of each protein was loaded in SDS-PAGE along with SeeBlue pre-stained protein marker (Invitrogen, USA). (A) *Bm*-RAL-2; (B) *Bm*-103; (C) *Bm*-RAL-2—*Bm*-103 fusion protein.

The DNA coding for *Bm*-103 without the C-terminal transmembrane domain was codon optimized and then synthesized by GenScript (Piscataway, NJ, USA) and subcloned in-frame into the yeast expression vector pPICZαA (Life Technologies, Carlsbad, CA, USA) with *EcoR*I/*Xba*I sites. Recombinant *Bm*-103 with 6xHis-tag at the C-terminus was expressed in *Pichia pastoris* X-33 under induction with 0.5% methanol and purified with immobilized metal affinity chromatography (IMAC) as described previously [19], [25]. *Bm*-103 is shown on a SDA-PAGE gel stained with Coomassie Blue with molecular weight of 14.5 KDa (Fig 1).

#### *Bm*-RAL-2

The *B*. *malayi*, *Bm*-RAL-2 (GenBank accession# XP_001900036) is a homolog of *Ov*-RAL-2 with an amino acid identity of 62%. The amino acid sequence comparison between *Bm*-RAL-2 and *Ov*-RAL-2 and the phylogenetic analysis of *Bm*-RAL-2 with other nematode homologs are shown as supplementary figures (Fig S3 A–B in S1 Dataset). *Ov*-RAL-2 was first shown to be a surface antigen in *O*. *volvulus* adult worm [20, 21] that induced protective immunity in an *O*. *volvulus* mouse model [19]. Pfam database search found it contained a DUF148 domain with unknown function. Immunoelectron microscopy studies were used to localize *Bm*-RAL and *Ov*-RAL in *B*. *malayi* and *O*. *volvulus*, respectively, using monospecific human anti-*Ov*-RAL-2 antibodies [24] that cross reacted with the *Bm*-RAL-2 protein. In *O*. *volvulus*, *Ov-*RAL-2 was not only localized in the hypodermis and cuticle of adult female worm as previously shown, but also to the surface of L3 larvae and MF as well as the glandular esophagus of L3s (Fig S4 in S1 Dataset). Similarly, *Bm*-RAL-2 was found to be localized in the hypodermis and cuticle of adult female worm, but it also newly shown that it is localized to the surface of L3 larvae and MF as well as the glandular esophagus of L3s (Fig S4 in S1 Dataset).

The DNA coding for *Bm*-RAL-2 without signal peptide was synthesized by GenScript (Piscataway, NJ, USA) and subcloned in-frame into *Escherichia coli* expression vector pET41a (EMDMillipore, Billercia, MA, USA) with the fusion GST deleted (NdeI/XhoI). Recombinant *Bm*-RAL-2 with 6xHis-tag at C-terminus was expressed under induction of 1 mM isopropyl β-D-1-thiogalactopyranoside (IPTG) and purified with IMAC as described previously [19], [26]. *Bm*-RAL-2 is shown on a SDA-PAGE gel stained with Coomassie Blue with molecular weight of 16 KDa (Fig 1).

#### *Bm*-103 –*Bm*-RAL-2 fusion protein

The *Bm*-103 and *Bm*-RAL-2 proteins were expressed as a fusion protein to study the protection of these two recombinant proteins in combination. The coding regions of *Bm*-103 and *Bm*-RAL-2 were fused using a flexible linker present in the *Necator americanus* protein *Na*-ASP-1 and which was shown by structural analyses to connect the two pathogenesis-related (RP) domains [25, 27]. The linker has no specific function but facilitates flexible hanging of the two pathogenesis-related domains and hence was chosen to connect *Bm*-103 and *Bm*-RAL-2 in this study. The fusion recombinant plasmid DNA was cloned into *E*. *coli* expression vector pET41a (EMD Millipore, USA) with GST knockout and recombinant fusion protein was expressed and purified as described above. The expressed *Bm*-103 –*Bm*-RAL-2 fusion protein had a correct predicted molecular weight of 36 KDa based on the protein sequence with some small molecular weight degraded bands (Fig 1). Based on deduced amino acid sequences, the *Bm*-103 –*Bm*-RAL-2 fusion protein has 60% identity with the protective *Ov*-103 –*Ov*-RAL-2 fusion protein [19]. The amino acid sequence alignment of fusion proteins, *Bm*-103 –*Bm*-RAL-2 and *Ov*-103 –*Ov*-RAL-2 are shown as supplementary figures (Fig S5 in S1 Dataset).

### Ethics statement

Animals were handled according to the NIH guidelines for animal experimentation and the animal experimental protocols were approved by the Louisiana State University Institutional Animal Care and Use Committee under the protocol number: 12–037. The animal care and use protocol adhered to the “Guide for the Care and Use of Laboratory Animals (the Guide)” published by the National Research Council, USA.

### Animals and parasites

Eight to ten week old male Mongolian gerbils (*Meriones unguiculatus*) (Charles River, USA) were used in all the vaccination experiments. Animals were housed in cages containing wood shaving bedding with 5 gerbils per cage. They were fed autoclavable rodent chow and given water *ad libitum*. Cages were kept in animal housing rooms in Louisiana State University School of Veterinary Medicine (LSU-SVM) that were pathogen-free with controlled temperature, humidity and light cycle conditions. The life cycle of *B*. *malayi* is being maintained at the LSU-SVM using Mongolian gerbils as the definitive host for the parasite and the Black Eyed Liverpool strain of *Aedes aegypti* as the intermediate host and vector. *B*. *malayi* L3 were recovered from infected mosquitoes on day 14 post blood meal using the previously described Baermann technique [28]. In two experiments (5 and 9, Table 1), the *B*. *malayi* L3s were obtained from Filariatech Inc, Athens, Georgia, USA. The L3s from this source are used by many investigators and the recoveries from gerbils inoculated with these L3s are comparable with those obtained when we were using L3s from our own colony. The L3s from Filariatech were produced by feeding *A*. *aegypti* on microfilareamic cats and harvested using the Baermann technique as described above. L3s were shipped in 50 ml falcon tubes containing RPMI medium at room temperature via an overnight express courier service from Athens, GA to LSU-SVM. Once received, L3s were washed three times with fresh RPMI medium and then used immediately for SC infection.

**Table 1:** Vaccination experiments conducted with *Brugia malayi* (*Bm*) recombinant proteins *Bm*-RAL-2, *Bm*-103, the *Bm*-103—*Bm*-RAL-2 fusion protein and with *Bm*-103 + *Bm*-RAL-2 concurrently.

Experiment Number	Vaccine	Necropsy—days post-infection (dpi)
**1**	*Bm*-RAL-2	42
**2**	*Bm*-103	42
**3**	*Bm*-RAL-2	120
**4**	*Bm*-103	120
**5**	*Bm*-RAL-2; *Bm*-103	150
**6**	*Bm*-RAL-2 + *Bm*-103 (Concurrent)	90
**7**	*Bm*-RAL-2—*Bm*-103 (Fusion)	90
**8**	Concurrent; Fusion	90
**9**	Concurrent; Fusion	150

### Experimental protocol for vaccination and L3 challenge

Multiple vaccination experiments were performed using recombinant *Bm*-103, *Bm*-RAL-2, *Bm*-103—*Bm*-RAL-2 fusion protein and with concurrent injection of both alum-formulated proteins but in separate sites (concurrent; *Bm*-103 + *Bm*-RAL-2). Necropsies were performed at different time points depending on the objective of the study. All the vaccination experiments consisted of at least 2 groups, an antigen vaccinated group(s) and an adjuvant control group with 10 animals in each group. Alum was used in all cases as the adjuvant, and the antigen-adjuvant formulation and vaccination protocols were performed as described previously except that the route of vaccination was intraperitoneal [19]. This is the most common and successful route of vaccination in terms of inducing protective immunity in *B*. *malayi*–rodent models [17, 29–36]. The gerbils in antigen vaccinated groups received 25 μg of recombinant protein in 0.1 ml of Tris Buffered Saline (TBS) formulated with 0.1 ml of 1:5 Rehydragel LV (alum) in TBS (General Chemical, Parsippany, NJ, USA). Gerbils in the adjuvant control groups received alum in TBS only. Gerbils were vaccinated intraperitoneally 3 times (V1, V2, and V3) at 2 week intervals. Antigen-adjuvant formulation was same as above for concurrent vaccinations of individual *Bm*-103 and *Bm*-RAL-2 antigens, but the gerbils in the vaccinated groups received two separate injections of alum-formulated *Bm*-103 and alum-formulated *Bm*-RAL-2 at different locations in the intraperitoneal cavity; gerbils in the adjuvant control group received equivalent amount of alum alone. Hence, the gerbils vaccinated with two antigens (concurrent) and their adjuvant controls received twice the amount of alum as the gerbils vaccinated with only one of the antigens. Two weeks after the third vaccination (Post-V3), all gerbils were challenged with 100 *B*. *malayi* L3 larvae subcutaneously in the medial surface of the left thigh.

A summary of the experiments conducted is shown in Table 1. In experiments 1 and 2, we tested the vaccine efficacy of *Bm*-RAL-2 and *Bm*-103 individually, respectively, and the necropsies were performed at 42 dpi. Necropsy at 42 dpi allowed for recovery of adult worms in a short period of time. Experiments 3 and 4 were similar to experiments 1 and 2, respectively, except the necropsies were performed at 120 dpi. This time point allowed a much easier recovery of the adult worms due to their increased size. In experiment 5, the vaccine efficacy of *Bm*-RAL-2 and *Bm*-103 was tested in parallel in a single experiment using the same adjuvant control group while the necropsies were performed at 150 dpi. Necropsies at 150 dpi also allowed for studying the effect of vaccination on microfilariae levels in gerbil blood, and on embryogenesis in the fertile female worms. In experiment 6, we tested the vaccine efficacy of concurrent vaccination with *Bm*-RAL-2 and *Bm*-103 and the necropsies were performed at 90 dpi. Experiment 7 tested the vaccine efficacy of *Bm*-RAL-2—*Bm*-103 fusion protein and necropsies were performed at 90 dpi. Experiment 6 and 7 were repeated together in experiment 8. Experiment 9 was similar to experiment 8 except that the necropsies were performed at 150 dpi, and the embryogenesis in the surviving female worms was studied using the embryogram method.

Necropsies were performed as previously described [37]. Adult worms were collected from the heart and lungs, right and left spermatic cord lymphatics, right and left testes and other lymphoid organs including the right and left popliteal lymph nodes, right and left renal lymph nodes, ilio-lumbar vessels, right and left sub-inguinal and iliac lymph nodes and vessels and the peritoneal cavity. Worm recoveries were also examined by site of recovery and therefore the distribution of worms in various organs was also recorded. Worms recovered from the spermatic cord, testes and lymph nodes were grouped as lymphatic organ worms, and worms recovered from the heart and lungs were grouped as heart and lungs worms. The worms recovered from viscera/body cavity were not assigned to either of these two groups, as their site of origin was unknown. The worm recovery data for heart & lungs and lymphatics tissue is presented as mean ± SD of worms within the organs. Additionally, the female: male ratio was calculated for each experiment to determine whether the vaccination with *Bm-*RAL-2, *Bm*-103, fusion protein, or concurrent vaccine affected male and female worms differently.

### Measurement of total IgG response by ELISA

Antigen-specific IgG responses to the each of the corresponding recombinant protein were measured in sera collected after third vaccination (post-V3) and prior to challenge infection using ELISA and following a protocol described previously [36]. ELISA plates were coated with recombinant *Bm*-RAL-2, *Bm*-103 or *Bm*-RAL-2—*Bm*-103 fusion protein and were tested with sera of the individual gerbils vaccinated with the respective antigens. *Bm*-RAL-2, *Bm*-103 and *Bm*-RAL-2—*Bm*-103 fusion protein coated plates were also tested with serum collected from gerbils immunized with *Bm*-RAL-2 + *Bm*-103 concurrently. The endpoint titers were calculated as the serum dilution from experimentally vaccinated animals that had an O.D. reading of 0.06 which is the O.D. recorded for highest control serum dilution (1:1000). Serial dilutions of gerbil serum were made and total antigen-specific IgG responses are reported as median with 25% and 75% percentile of endpoint titers of the group because of few extreme outliers in the data set and the median expresses a better middle value (central tendency) in a set of data than the mean/average.

### Embryogram and microfilariae count

To study the effect of vaccination on embryogenesis, embryograms were performed on the surviving female worms to enumerate the developing embryonic stages *in utero* (eggs, embryos, developing microfilariae and stretched microfilariae) as described previously [36]. Adult female worms recovered 150 dpi (Experiments 5 and 9) were used for this purpose. Additionally, in experiment 5 and 9, the number of circulating microfilariae in 500 μl of peripheral blood of individual gerbils at the time of necropsy was determined using a Knott’s assay [38], [39].

### *In vitro larval* killing assay

An L3 *in vitro* killing assay was performed as described previously [36] with slight modifications. Pooled sera from gerbils immunized with *Bm*-RAL-2, *Bm*-103, *Bm*-RAL-2—*Bm*-103 fusion or *Bm*-RAL-2 + *Bm*-103 were used in the assay. Based on availability of sufficient quantities of sera, only the sera samples collected post V2 were used in the *in vitro* killing assay using 1:20 dilution of serum and 2 x 10^5^ thioglycolate stimulated peritoneal exudate cells (PEC) from naïve gerbils. Performed in quadruplicate, 20 *B*. *malayi* L3s were cultured under different treatment conditions and their survival was scored based on motility and morphology at 48 hrs post-treatment. Worms that were completely immotile with granulated and disrupted internal tissues were considered dead. The treatment groups were as follows: RPMI media control; PEC only; pre-immune serum with PEC; alum serum with PEC; anti-*Bm*-RAL-2 serum alone; anti-*Bm*-103 serum alone; anti-(*Bm*-RAL-2—*Bm*-103) serum alone; and anti-(*Bm*-RAL-2 + *Bm*-103) serum alone; anti-*Bm*-RAL-2 serum with PEC; anti-*Bm*-103 serum with PEC; anti-(*Bm*-RAL-2—*Bm*-103) serum with PEC; and anti-(*Bm*-RAL-2 + *Bm*-103) serum with PEC.

### Statistics

All the vaccination experiments consisted of 10 gerbils per group and the statistical significance between antigen-vaccinated gerbils and alum only controls were analyzed by Mann-Whitney U test using GraphPad Prism 6 (GraphPad Software, San Diego, California USA). Protective immunity induced by vaccination was determined by two metrics, percentage worm burden reduction and host protection, as described earlier [19]. Percentage reduction was calculated by subtracting the average number of worms recovered in antigen-vaccinated gerbils from the average number of worms recovered from the control gerbils divided by average worms recovered from the control gerbils, and then multiplied by 100. This metric is a measure of protection efficacy in a vaccine group based on worm recovery. Host protection determines what percent of the vaccinees within the vaccination group benefited from the vaccination and had less worm burden than the control group. Statistical analysis of host protection was performed using bootstrap analysis. The bootstrap sample mean was estimated based on the worm burden below the lower boundary of the 95% confidence interval of the control group (Host Protection). Then a kernel density estimate of the vaccine group (N = 10) was calculated and the percentage below the bootstrap 95% confidence interval was calculated. The R package "boot" was used for the analysis [40–42].

## Results

### Vaccination with *Bm*-RAL-2 and *Bm*-103 individually, concurrently or as a fusion protein induces protective immunity against *B*. *malayi* infection in Mongolian gerbils

**Fig 2:**
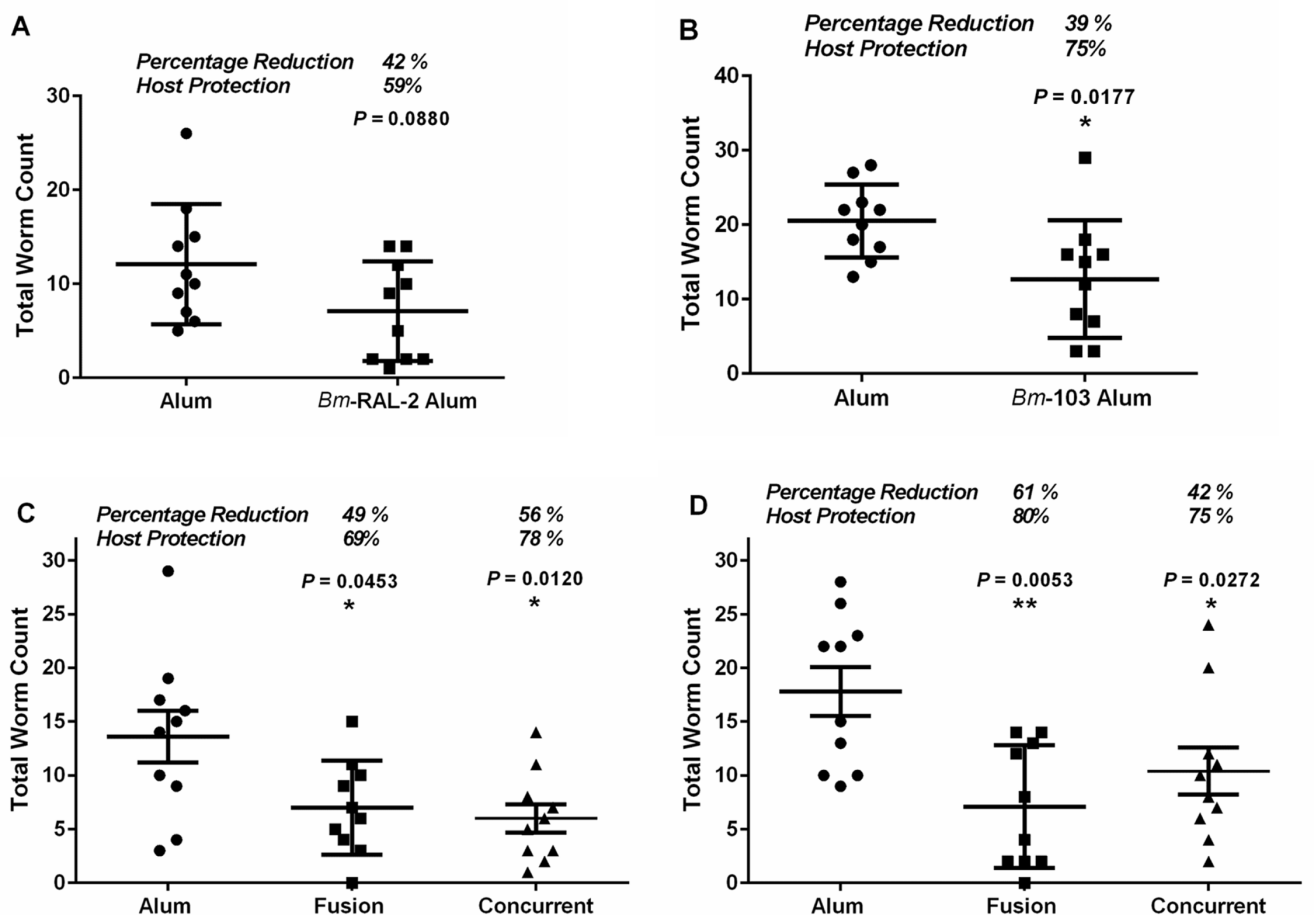
Effect of vaccination with *Bm*-RAL-2 and *Bm*-103 individually, concurrently and as a fusion protein on the development of protective immunity manifested as % reduction in worm burdens and % host protection against infection with *Brugia malayi* L3s in Mongolian gerbils. (A) Vaccination with *Bm*-RAL-2 (42 dpi); (B) Vaccination with *Bm*-103 (42 dpi); (C) Vaccination with *Bm*-RAL-2—*Bm*-103 fusion protein or with *Bm*-RAL-2 + *Bm*-103 concurrently (90 dpi); (D) Vaccination with *Bm*-RAL-2—*Bm*-103 fusion protein or *Bm*-RAL-2 + *Bm*-103 concurrently (150 dpi). Each dot represents the total number of adult worms recovered from an individual animal. The lines represent mean with standard deviation. *P* ≤ 0.05 denotes a statistically significant difference in total worm recovery (percent reduction) between vaccinated and alum control group, Mann–Whitney U Test, GraphPad Prism 6.

A summary of the results of all the performed experiments is presented in Table 2. Detailed data from selected experiments are shown in each category and described below (Fig 2).

**Table 2:** Vaccine efficacy, percentage reduction in worm recovery and host protection induced by vaccination of gerbils with *Bm*-RAL-2 and *Bm*-103 individually, concurrently or as a fusion protein.

Vaccine	Experiment Number	Necropsy dpi	% Reduction^a^	*P* value	% Host Protection^b^
***Bm*-RAL-2**	1	42	42	0.0880	59
	3	120	22	0.3826	46
	5	150	46	0.0131	77
***Bm*-103**	2	42	39	0.0177	75
	4	120	34	0.0366	69
	5	150	22	0.3229	50
**Concurrent**	6	90	61	0.0072	76
	8	90	56	0.0120	78
	9	150	42	0.0272	75
**Fusion**	7	90	51	0.0398	66
	8	90	49	0.0453	69
	9	150	61	0.0053	80

a, Percentage reduction in worm burden was calculated as follows: % reduction = (average number of worms surviving in control group–average number of worm surviving in vaccinated group)/average number of worm surviving in control group) × 100.

b, Bootstrap analysis of host protection. The measure of host protection determines the percentage of vaccinees within the vaccinated group benefited from the vaccination (worm burden below the lower boundary of the 95% confidence interval).

### Vaccination with *Bm*-RAL-2

In experiment 1, gerbils were vaccinated with *E*. *coli* expressed recombinant *Bm*-RAL-2 formulated with alum and then challenged subcutaneously with 100 *B*. *malayi* L3s. To more quickly investigate the effect of vaccination, necropsy was performed 42 dpi. *Bm*-RAL-2 induced 42% reduction in worm burden and a 59% host protection when compared with alum control (Fig 2A). The effect of vaccination with *Bm*-RAL-2 on long-term (120 and 150 dpi) *B*. *malayi* infection in gerbils was 22% and 46% reduction of worm burden and 46% and 77% host protection, respectively, when compared to alum controls (Table 2). The worm reduction of 46% at 150 dpi was statistically significant when compared with alum controls.

### Vaccination with *Bm*-103

Experiment 2 was similar to experiment 1 except that a *P*. *pastori* expressed recombinant *Bm*-103 protein was used. Vaccination with alum-formulated *Bm*-103 induced a significant worm burden reduction of 39%, and a 75% of host protection when compared with the alum control group (Fig 2B). The effect of vaccination with *Bm*-103 on long-term (120 and 150 dpi) *B*. *malayi* infection of gerbils was a 34% and 22% reduction of worm burden and 69% and 50% host protection, respectively, when compared to alum controls (Table 2).

### Vaccination with *Bm*-103 and *Bm*-RAL-2 concurrently or as fusion protein

In experiments 6–9, we tested the hypothesis that vaccination with *Bm*-RAL-2 and *Bm*-103 concurrently or as a fusion protein would enhance protection. Vaccination with *Bm*-RAL-2 + *Bm*-103 concurrently induced a significant reduction of 56–61% in worm burden and a host protection of 76–78% when compared with alum control groups (90 dpi) (Table 2). Vaccination with the fusion protein induced a significant 49–51% reduction in worm burden and a host protection of 66–69% when compared with alum control groups (90 dpi). A detailed outcome of experiment 8 is illustrated in Fig 2C.

Experiment 9 was a repeat of experiment 8 except that the necropsy was performed at 150 dpi. In comparison to alum controls, vaccination with *Bm*-RAL-2 + *Bm*-103 concurrently and as a fusion protein induced a significant reduction in worm burdens of 42% and 61% respectively, and conferred a host protection of 75% and 80%, respectively (Fig 2D, Table 2).

When the distribution patterns of the recovered worms in the heart & lungs and in the lymphatic organs was compared between the vaccinated and the alum control groups it appeared that in most cases the reduction in worm burden was not statistically significant in the separate organs, indicating that the homing of the surviving worms had not been specifically impacted by the vaccine as previously shown when gerbils were vaccinated with *Bm*-CPI-1 and *Bm*-CPI- 2 (37). The impact of the vaccines was reduction in the total number of worms recovered regardless of where they were recovered from (S1 Table). In few of the experiments (Experiment 5, *Bm*-RAL-2; Experiment 6, concurrent vaccination; and Experiment 9, vaccinated with a fusion protein) in which a significant reduction of worm burden in a defined anatomical location was observed, it was not reproduced in the repeated experiments, and therefore might have arisen accidentally (S1 Table).

The female: male ratio data of all the experiments is shown in S2 Dataset (Fig S1 –S9 in S2 Dataset). The female: male ratio was between 0.81 and 1.65 in alum control groups of gerbils. The ratio was not significantly different in *Bm*-RAL-2 vaccinated gerbils. In 2 of 3 *Bm*-103 vaccination experiments the female: male ratio was significantly greater than alum controls (Fig S4 and S5 in S2 Dataset, *P* = 0.0094 and *P* = 0.0337). Vaccination with either the fusion protein or both proteins in a concurrent vaccination did not alter the female: male ratio.

### IgG responses induced by vaccination with *Bm*-RAL-2 and *Bm*-103 individually, concurrently or as a fusion protein

**Fig 3:**
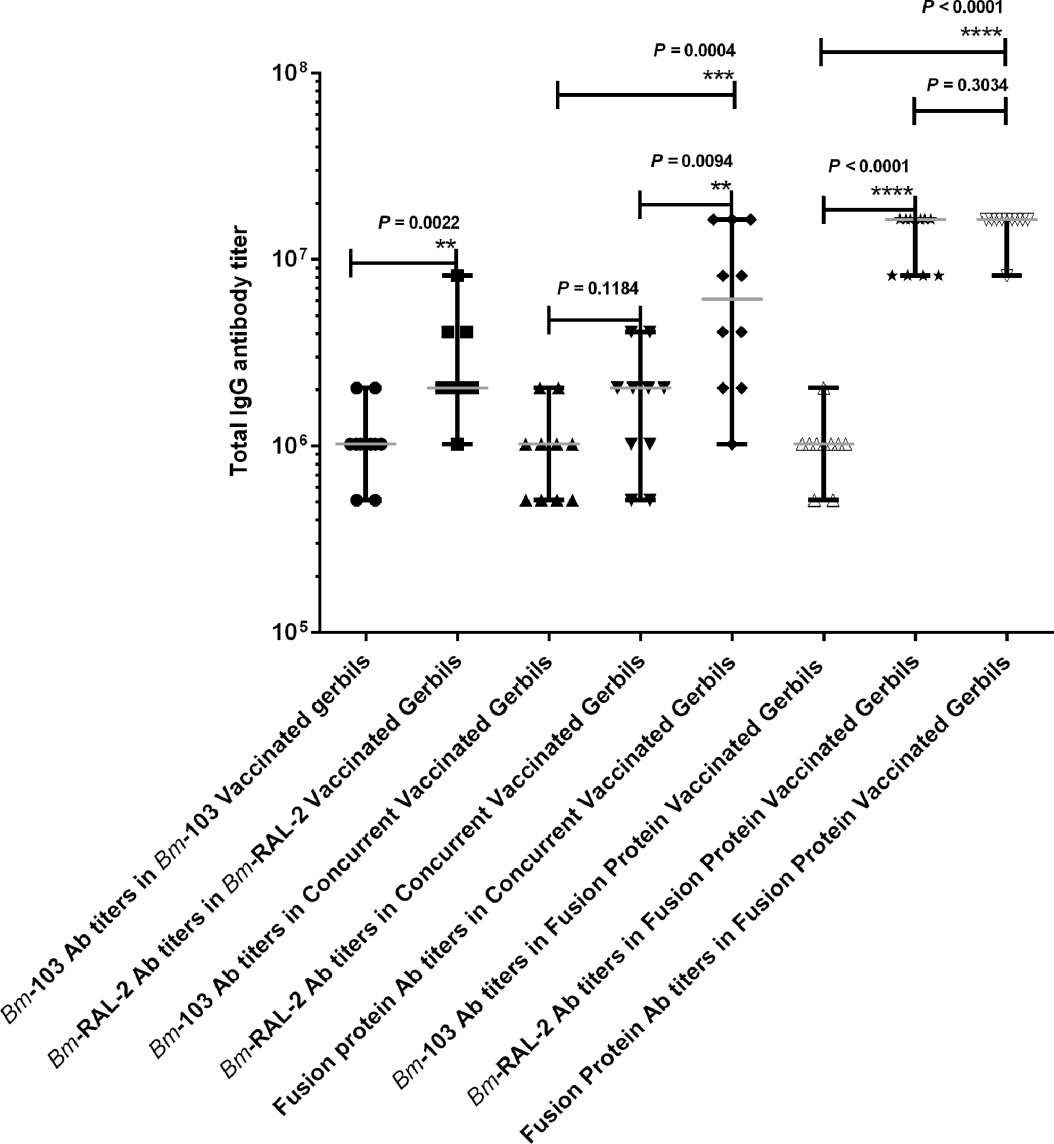
Total IgG endpoint titers specific to *Bm*-103, *Bm*-RAL-2 or the fusion protein in gerbils vaccinated (N = 10) with *Bm*-RAL-2 and *Bm*-103 individually, concurrently or as a fusion protein. Each dot represents IgG endpoint titer from an individual animal. The lines represent median with maximum and minimum range. Asterisk/s denotes a statistically significant difference in IgG endpoint titers between analyzed groups, Mann–Whitney U Test, GraphPad Prism 6 (*, *P* ≤ 0.05; **, *P* ≤ 0.01; ***, *P* ≤ 0.001; ****, *P* ≤ 0.0001).

The antigen-specific IgG endpoint titers in animals vaccinated three times with *Bm*-RAL-2 and *Bm*-103 individually, concurrently or as a fusion protein are shown in Fig 3 and in Table 3. Fig 3 shows endpoint titers in each vaccinated gerbil within the group whereas Table 3 specifically shows the median with 25% and 75% percentile for the vaccinated group. Vaccination with all the vaccine antigens and their combinations elicited strong antigen-specific IgG responses to the corresponding antigens. The antibody data presented in Fig 3 and Table 3 are from experiments terminated 150 dpi (Experiments 5 and 9) and are representative of all the other experiments. The IgG endpoint titers in animals vaccinated with *Bm*-RAL-2 were significantly and 4 times higher than that in animals vaccinated with *Bm*-103 (*P* = 0.0022). In gerbils vaccinated with *Bm*-RAL-2 + *Bm*-103 concurrently, the *Bm*-RAL-2-specific IgG endpoint titer was higher than that of anti-*Bm*-103, though this was not statistically significant (*P* = 0.1184). Interestingly, when the serum from animals vaccinated with *Bm*-RAL-2 + *Bm*-103 were tested against the *Bm*-RAL-2—*Bm*-103 fusion protein, the IgG endpoint titer was significantly and 4 or 8 times higher than those specific to the individual antigens, *Bm*-RAL-2 or *Bm*-103, respectively (*P* = 0.0094 and *P =* 0.0004). Serum from animals vaccinated with the fusion protein also reacted with the individual antigens, *Bm*-RAL-2 and *Bm*-103; with the *Bm*-RAL-2-specific IgG endpoint titer being significantly and 4 times higher than that of anti-*Bm*-103 (*P* < 0.0001). The IgG endpoint titers against the fusion protein were also significantly and 8 times higher than those of the anti-*Bm*-103 (*P =* 0.0001), but not of those of anti-*Bm*-RAL-2 (*P =* 0.3034).

**Table 3:** IgG endpoint titers in gerbils vaccinated with *Bm*-RAL-2 and *Bm*-103 individually, concurrently or as a fusion protein at 2 weeks after 3^rd^ vaccination (experiments 5 and 9).

Vaccinating Antigen	Antigen-specific endpoint titers		
	Median		
	(25%; 75% Percentile)		
	*Bm*-RAL-2	*Bm*-103	Fusion protein
***Bm*-RAL-2**	2,048,000		
	(2,048,000; 4,096,000)		
***Bm*-103**		1,024,000	
		(896,000; 1,280,000)	
**Fusion**	16,380,000	1,024,000	16,380,000
	(8,192,000;16,380,000)	(1,024,000;2048,000)	(16,380,000;16,380,000)
**Concurrent**	2,048,000	1,024,000	6,144,000
	(896,000; 2,560,000)	(512,000; 1,280,000)	(2,048,000; 16,380,000)

To determine if any difference in antibody levels among the vaccinated gerbils had an effect on worm recovery in each of the gerbils, a correlation analysis of antibody levels and worm numbers was performed using the antigen-specific optical density obtained at 1:32,000. This dilution was chosen because the magnitude of difference in antibody levels was highest at this dilution between gerbils with high and low antibody titer. No significant relationships were detected between the antibody titers of individual gerbils vaccinated with *Bm*-103, *Bm*-RAL-2 + *Bm*-103 concurrently or as a fusion protein and the number of worms recovered from those individual animals. However, there was a correlation between higher antibody titers to *Bm*-RAL-2 in *Bm*-RAL-2 vaccinated gerbils and higher worm recoveries in those animals (Table 4). Nonetheless, even in this group the antibody titers did not correlate with the reduced number of worms recovered and protection.

**Table 4:** Correlation analysis between antibody levels and worm recovery.

Vaccinating Antigen	Correlation analysis between antibody levels and worm recovery	
	*R* Value	*P* Value
***Bm*-RAL-2**	0.6441	0.0444
***Bm*-103**	-0.1135	0.7548
**Fusion**	0.1226	0.7359
**Concurrent**	0.1002^a^	0.7838
	0.2670^b^	0.4558

^a^ Correlation analysis between IgG titers to *Bm*-RAL-2 and worm recovery in *Bm*-RAL-2 + *Bm*-103 concurrently vaccinated gerbils.

^b^ Correlation analysis for IgG titers to *Bm*-103 and worm recovery in *Bm*-RAL-2 + *Bm*-103 concurrently vaccinated gerbils.

### Effect of vaccination with *Bm*-103 and *Bm*-RAL-2 individually, concurrently or as a fusion protein on circulating microfilariae and embryogenesis in female worms

**Fig 4:**
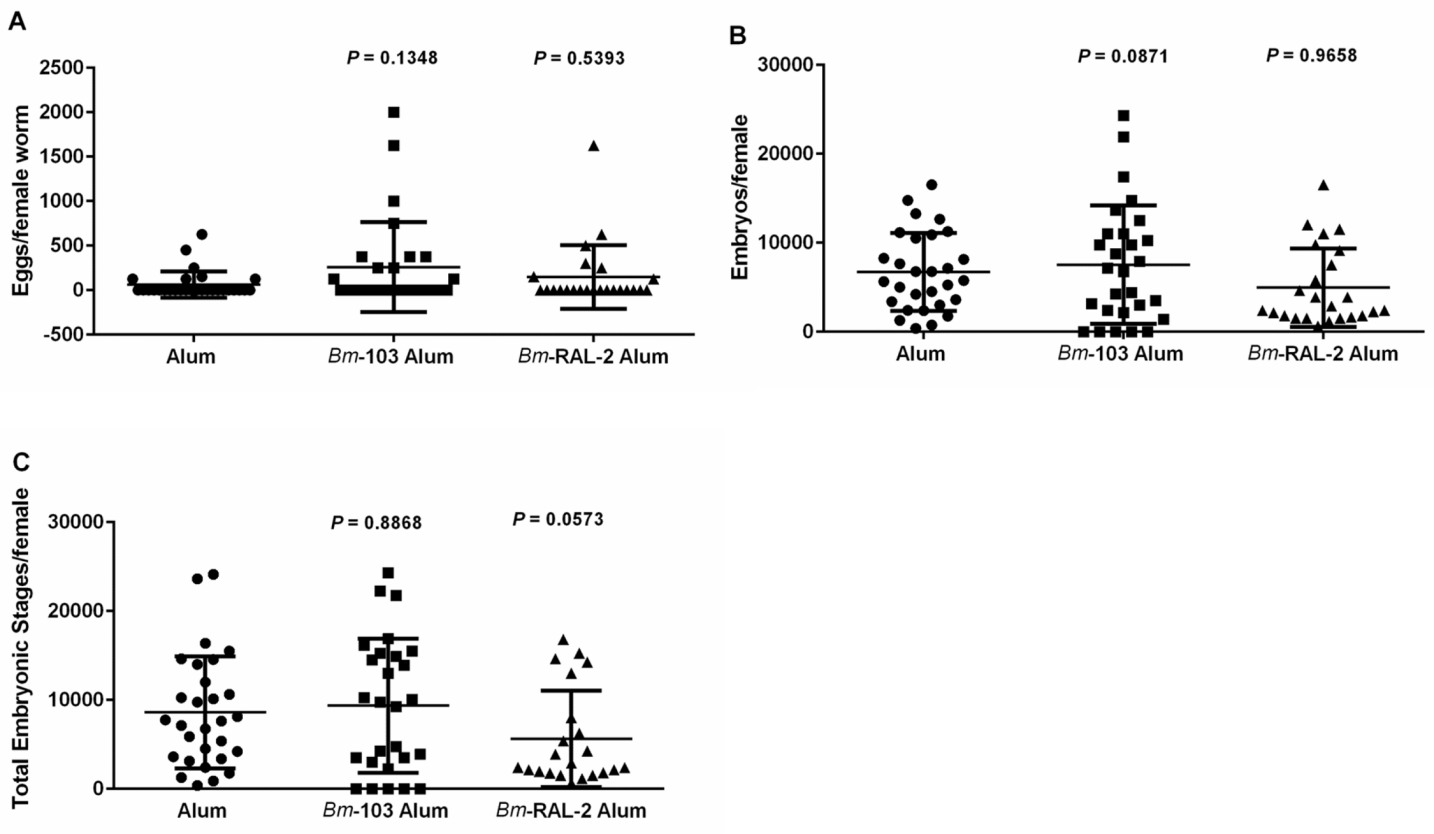
Embryogram data of *B*. *malayi* female worms recovered 150 dpi from alum control, *Bm*-RAL-2 and *Bm*-103 vaccinated gerbils. (A) Eggs/female worm; (B) Embryos/female worm; (C) Total embryonic stages/female worm. Statistical significance was determined by Mann–Whitney U Test using GraphPad Prism version 6, *P* ≤ 0.05. The line represents mean with standard deviation.

To measure the levels of microfilariae in the blood, as well as to examine fertility of the surviving female worms, necropsies of gerbils vaccinated with *Bm*-103 and *Bm*-RAL-2 individually, concurrently or as fusion protein were performed at 150 dpi. Although there was a reduction in microfilariae counts in the gerbils vaccinated with alum-formulated *Bm*-103, the percent reduction was not statistically significant when compared with alum control groups (56%, *P* = 0.2561; Fig S1 in S3 Dataset). However, there was a significant reduction in microfilariae counts in gerbils vaccinated with alum-formulated *Bm*-RAL-2 (63%, *P* = 0.0410; Fig S1 in S3 Dataset). When embryograms were performed on the recovered female worms in vaccinated gerbils and the alum control, it appeared that in *Bm*-103 vaccinated gerbils there was no significant difference in the number of eggs, embryos and the total embryonic stages in the female worms (Fig 4A–4C). In *Bm*-RAL-2 vaccinated gerbils, however, there was a substantial reduction (*P* = 0.0573) in the total embryonic developmental stages within the female worms collected from *Bm*-RAL-2 vaccinated animals when compared with the alum control (Fig 4A–4C), which possibly explains the significant decrease in the microfilariae in the blood seen in this group.

**Fig 5:**
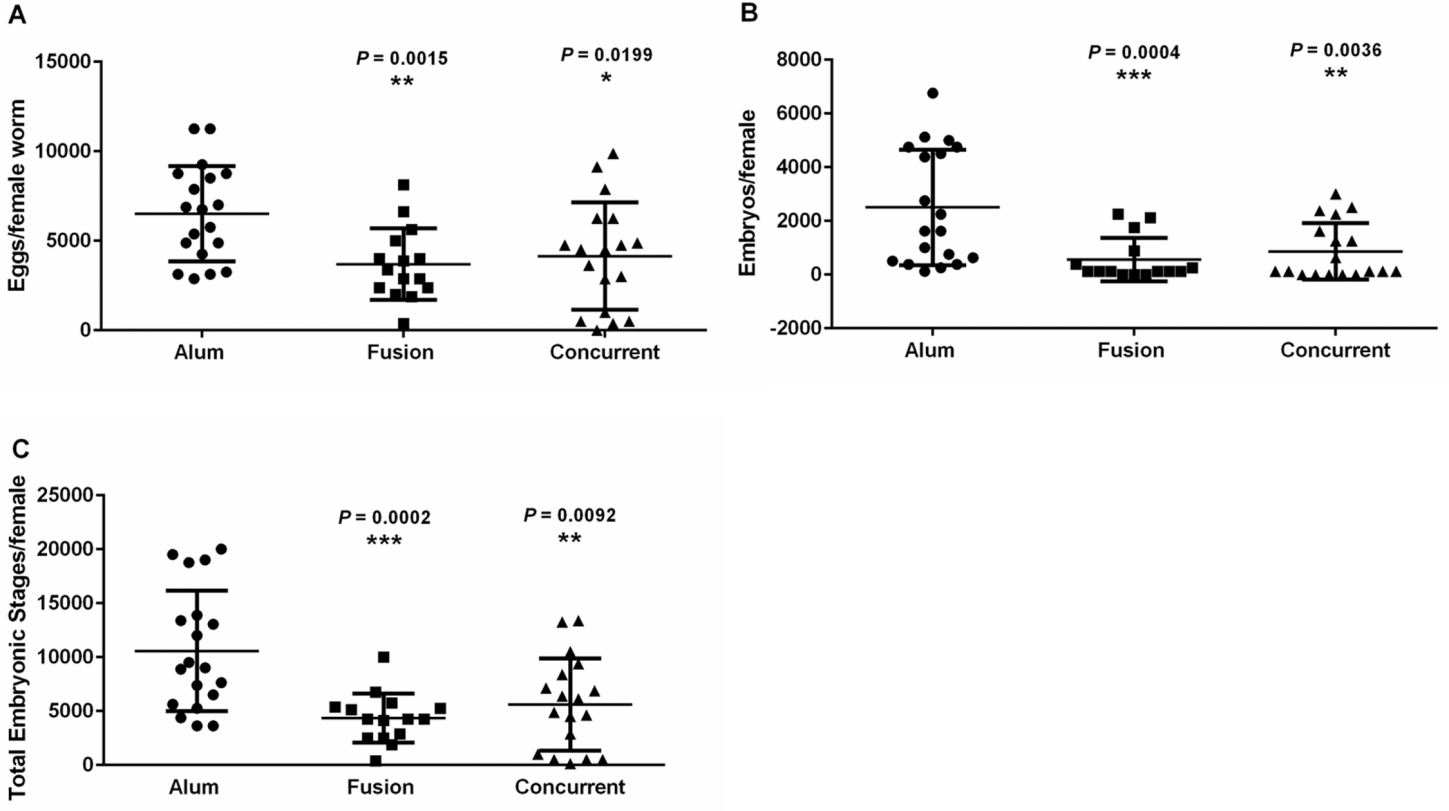
Embryogram data of *B*. *malayi* female worms recovered 150 dpi from alum control, *Bm*-103 + *Bm*-RAL-2 and *Bm*-103—*Bm*-RAL-2 vaccinated gerbils. (A) Eggs/female worm; (B) Embryos/female worm; (C) Total embryonic stages/female worm. Statistical significance was determined by Mann–Whitney U Test using GraphPad Prism version 6, asterisks denotes a significant difference between the alum control and the vaccinated group, *P* ≤ 0.05. The line represents mean with standard deviation.

Notably, although the microfilariae levels in gerbils vaccinated with *Bm*-103 + *Bm*-RAL-2 concurrently or as a fusion protein 150 dpi were similar to those found in the control groups (Concurrent vs control, *P* = 0.1708; fusion vs control, *P* = 0.1834; Fig S2 in S3 Dataset), the embryogram data showed a significant reduction in eggs, embryos and the total embryonic stages within the female worms recovered from the vaccinated gerbils when compared with the alum control groups (Fig 5A–5C).

### *In vitro* killing assay

*B*. *malayi* L3 were cultured in the presence of PEC with or without serum from animals vaccinated with *Bm*-103 and *Bm*-RAL-2 individually, concurrently or as a fusion protein. Pre-immune serum or serum from alum control groups served as the control sera. L3 survival was scored 48 hrs post-treatment and the results are presented in Table 5. All the L3 survived in the control serum groups including sera from vaccinated groups without PEC. In comparison, a small but consistent killing was observed when the L3 were cultured in the presence of PEC and anti-*Bm*-103 serum (31%) or with anti-*Bm*-RAL-2 serum (15%). Serum from gerbils vaccinated with *Bm*-RAL-2 + *Bm*-103 concurrently or with the fusion protein induced killing of 16 and 20%, respectively (Table 5). Only the L3s that were completely immotile with severely granulated and disrupted internal tissues were considered as dead and the slow moving larvae with PEC attached to its surface were not scored as dead worms. Hence, a stringent scoring system was used to evaluate the killing assay, which might account for the reduced percentage of L3 killing reported in this study. Irrespectively, overall more L3s were affected by PEC plus antigen-specific serum. To illustrate the *in vitro* killing, a video clip of *B*. *malayi* L3 cultured in the presence of alum serum and PEC (S1 Video) and a video clip of *B*. *malayi* L3 cultured in the presence of anti-*Bm*-103 serum and PEC (S2 Video) are presented as supporting data. In the future, we might adopt a less stringent approach that also counts the partially non-motile larvae as dead if cells are highly adhered to their surface and as shown in S2 Video.

**Table 5:** *Brugia malayi* L3 larvae *in vitro* killing assays.

Treatment	Percentage of L3 killing (%)
**Media Control**	0
**PEC alone**	0
**Pre-immune serum + PEC**	0
**Alum serum + PEC**	0
**Anti-*Bm*-RAL-2 serum**	0
**Anti-*Bm*-103 serum**	0
**Anti-(*Bm*-RAL-2—*Bm*-103) serum**	0
**Anti-(*Bm*-RAL-2 + *Bm*-103) serum**	0
**Anti-*Bm*-RAL-2 serum + PEC**	15
**Anti-*Bm*-103 serum + PEC**	31
**Anti-(*Bm*-RAL-2—*Bm*-103) serum + PEC**	20
**Anti-(*Bm*-RAL-2 + *Bm*-103) serum + PEC**	16

PEC, peritoneal exudate cells (Mongolian gerbils).

## Discussion

Previous studies have shown that protective immunity against *Brugia spp* is readily induced by irradiated *Brugia* L3 in Rhesus monkeys [43], cats [44] and gerbils [45]. In addition, a wide variety of recombinant proteins of *B*. *malayi* have also been shown to provide some degree of protection ranging from 40% to 95% in worm burden reduction in various animal models (some reviewed by Morris et al., 2013 [19, 29–36]). However, the current data is the first report to demonstrate that alum-formulated *Bm*-103 or *Bm*-RAL-2 individually, concurrently or as a fusion protein induce significant protection against a subcutaneous *Brugia* L3 challenge infection that resembles the course of infection in humans [46]. The range of protection seen in the current study is consistent with previous reports of vaccination with other recombinant *Brugia* proteins, even though the studies vary in many ways including the character of the antigen, the adjuvant, the route of immunization, the route of challenge and the animal model used. A majority of the other vaccination experiments were performed using the mouse-diffusion chamber model or intraperitoneal infection (IP) of gerbils or southern multimammate mouse with *B*. *malayi* L3 [17], [31], [47–52]. Moreover, in most cases, the vaccination experiments were also not repeated to confirm consistency in inducing protective immunity by the vaccine candidates tested. Very few investigators [30], [32], [53] have used the subcutaneous route of infection of gerbils with *B*. *malayi* L3 as we report in the current study. This model more closely mimics the natural infection pattern in humans and is a more critical test for substantiating that the host protective immune response to such a challenge infection can be reproduced in humans. In this system L3s, the other larval stages and early adult stages must migrate through various connective tissues and locate suitable sites for development in the lymphatic system and the blood vascular system [46], [54]; all these stages may be targets of protective immunity as well [55]. Moreover, in this infection system, the adult stages mature and produce patent infections (microfilariae in the blood) allowing the measurement of the impact vaccination has on microfilariae burden as well, either through enumeration of circulating microfilariae in the blood or by measuring the embryonic development within the surviving fertile adult female worms by the embryogram assay [36]. The subcutaneous route of infection of gerbils with *B*. *malayi* L3 also induces a down regulation of the inflammatory and immune responses in the more chronic time of infection [56]. This state can be induced by all stages of the life cycle [57], and is correlated with the level of microfilaremia [58]. This condition mimics the microfilaremic-hypo-responsive state seen in human lymphatic filariasis [59]. Notably, in situations where the vaccination is protective but incomplete, as it is in most the cases, the surviving parasites might be capable of inducing a down regulation of the immune and inflammatory response [60], which may alter subsequent protective immune responses potentially complicating the model but also mimicking the natural situation, adding to the model’s value. Future experiments using an optimized vaccine could test this assumption experimentally in this gerbil infection model.

The objective of the present study was to test the efficacy of the *B*. *malayi Bm*-RAL-2 and *Bm*-103 proteins that are the homologs of two confirmed vaccine candidates of *O*. *volvulus*, *Ov*-RAL-2 and *Ov*-103. To this end, repeated vaccination experiments in the gerbil model were done to determine efficacy at different time points post infection. Additionally, the protective efficacy of administering *Bm*-RAL-2 and *Bm*-103 concurrently or as a fusion protein was tested at 90 and 150 dpi.

As was shown in the *O*. *volvulus* in mouse model [19], *Bm*-RAL-2 and *Bm*-103 repeatedly induced protection against *B*. *malayi* infection in gerbils. Vaccination with *Bm*-RAL-2 and *Bm*-103 induced protection ranging from 22–46% and 22–39%, respectively (Table 2). Vaccination with *Bm*-RAL-2 and *Bm*-103 concurrently and as a fusion protein induced a more consistent significant protective efficacy ranging from 42–61% and 49–61%, respectively (Table 2). It is important to note that while vaccination with the fusion protein or concurrently consistently resulted in a greater level of protection than when gerbils were vaccinated with the individual antigen, it was not possible to statistically compare these data because they were not directly compared in single experiments. Previously, enhanced level of protective immunity induced by a multivalent protein vaccine has also been demonstrated against *B*. *malayi* [35], [61–64]. However, increased levels of protection were not seen using similar bivalent immunizations with the homologous proteins in the *O*. *volvulus* mouse chamber model, which only can observe the effects of a vaccine on the development of L3 to L4 [19]. This further supports the advantage of testing the *O*. *volvulus* homologous proteins in the *B*. *malayi* gerbil model that allows enumerating the worm burden and effects on embryonic development in multiple ways. For example, analyses of female: male ratio were performed to investigate if the vaccination affected female and male worm survival differently. Vaccination with *Bm*-RAL-2, *Bm*-RAL-2—*Bm*-103 fusion protein or *Bm-*RAL-2 and *Bm*-103 concurrently did not have a significant impact on female: male ratio. These findings are consistent with a previous report where vaccination with irradiated L3 of *Acanthocheilonema viteae* and subsequent challenge with *A*. *viteae* L3 did not have an impact on female:male worm ratio in 3 different rodent hosts, gerbils, multimammate rats and golden hamsters [65]. However, vaccination with *Bm*-103 resulted in significantly higher female: male ratio in 2 out of the 3 experiments, suggesting that vaccination with *Bm*-103 might have affected male worms more than the female worms. It is unclear why this small but significant difference in male and female susceptibility to vaccination should occur. It did not occur in the gerbils vaccinated with the fusion protein or in concurrently vaccinated gerbils when *Bm*-103 was part of these vaccinations. It is possible that there is a differential expression of these proteins in both sexes and/or in different tissues but there is no evidence for this. Irrespective, there were other effects on the surviving female worms; reduced production of microfilariae and/or embryonic development within the worms.

One of the methods to measure fertility of female worms is to count the circulating microfilariae in the blood of vaccinated or adjuvant control gerbils. When compared with alum control groups, a significant reduction in MF was observed in gerbils vaccinated with *Bm*-RAL-2, whereas gerbils vaccinated with *Bm*-103, *Bm*-RAL-2—*Bm*-103 fusion protein or concurrently were not significantly different (Fig S1 –S2 in S3 Dataset). The reason for not being able to observe significant differences in levels of circulating MF in vaccinated vs. alum control groups could be related to the early time point that was chosen for necropsy (i.e. 150 dpi). The present experiments were more focused on measuring the efficacy of the vaccines against the development of the infections to the adult stages and this time point served this purpose well. In an independent project, we performed MF counts in *B*. *malayi* infected gerbils at different time points over the course of 3 years; higher levels of MF started to appear only after 200 dpi, which reached peak levels at 300 dpi (Fig S3 in S3 Dataset). Hence, long-term experiments in which we extend the necropsies to 300 dpi, when higher levels of MF are expected in the control groups, will allow us to determine the direct effect of vaccination with *Bm-*RAL-2 and *Bm*-103 as a fusion protein or co-administered concurrently on MF production *in vivo*. We plan to perform such experiments in the future. Regardless, necropsies at 150 dpi allowed us to conduct the embryogram assays in the surviving female worms, which gave us indirect data on the MF development. This demonstrated that although vaccination with *Bm*- RAL-2 and *Bm*-103 individually did not severely affect the fecundity of the surviving female worms, their fecundity was significantly reduced in gerbils vaccinated with *Bm*- RAL-2 and *Bm*-103 concurrently or as a fusion protein. Thus, vaccination with *Bm*- RAL-2 and *Bm*-103 concurrently or as a fusion protein induced significant levels of protection against a subcutaneous infection of Mongolian gerbils reducing the total worm burden as well as the fecundity of remaining surviving female worms by significantly reducing the numbers of eggs, embryos, and total embryonic stages within the female worms (Fig 4). Such an anti-fecundity effect of a vaccine may also support the reduction of circulating microfilariae in the endemic areas and thus hasten the interruption of transmission presently done only by MDA with drugs and thus meet the disease elimination targets. Mathematical modeling studies are underway to evaluate the impact that vaccination with a bi-valent vaccine administered concurrently or a fusion protein has on blocking transmission in areas where MDA is implemented [66].

The mechanism by which the immunity induced by the *Bm*- RAL-2 and *Bm*-103 administered concurrently or as a fusion protein affects embryogenesis in female worms is unknown. It may be related to the disruption of an unknown role of the proteins during embryogenesis. Interestingly, in both *B*. *malayi* and *O*. *volvulus*, both proteins are also expressed on the surface of microfilariae (Fig S2 and Fig S4 in S1 Dataset). *O*. *volvulus* antibodies against *Ov*-103 also kill microfilariae *in vitro* in the presence of neutrophils [22]. Recently, we reported that vaccination with a mutated *B*. *malayi* cysteine protease inhibitor (*Bm*-CPI-2M) also reduced the fecundity of female worms [28]. In *Caenorhabditis elegans*, the homolog of *Bm*-CPI-2M, cysteine protease inhibitor CPI-2a, was shown to have an essential regulatory role during oogenesis and fertilization [67], which suggested that *Bm*-CPI-2 could also have similar roles during embryogenesis in *B*. *malayi*. The functional properties of *Bm*-103 and *Bm*-RAL-2 are currently unknown. It is also possible a general morbidity is induced in the vaccinated animals against the female worms, which may be responsible for this effect. At this stage it is not possible to address the specific role(s) these proteins have during embryogenesis of *B*. *malayi* or how immunity induced by these vaccinations has reduced fecundity. Notably, vaccination can reduce fecundity in other helminths in addition to worm burden, including the vaccination of dogs with Ac-16, an immunodominant surface antigen from the hookworm *Ancylostoma caninum* that is a homolog of *Ov*-RAL-2 and *Bm*-RAL-2 [68–70].

In all of the vaccination experiments with *Bm*-RAL-2 and *Bm*-103 individually or as a fusion protein or concurrently a strong antigen-specific IgG response to the corresponding antigens was elicited. The responses against *Bm*-RAL-2 were always higher than those against *Bm-*103 regardless of if the protein was used for vaccination alone or in combination with *Bm-*103 when they were co-administered concurrently or as a fusion protein, pointing to the possibility that *Bm*-RAL-2 is a much stronger immunogen. Additionally, antibodies elicited in gerbils immunized with the fusion protein recognized both individual *Bm*-RAL-2 and *Bm*-103 recombinant proteins, suggesting that the expression of the *Bm*-RAL-2—*Bm*-103 fusion protein did not alter the individual protein structures or their functional properties. Although vaccination with alum-formulated *Bm*-RAL-2 or *Bm*-103 elicited strong antigen-specific IgG responses, sera taken from chronically infected gerbil sera (150 dpi) did not recognize either *Bm*-103 or *Bm*-RAL-2, suggesting that these proteins perhaps are not as highly immunogenic during a primary infection as other filarial proteins such as the *B*. *malayi* abundant larval transcript [21,71]. Correlation analysis between the numbers of worms recovered and the antigen-specific antibody endpoint titers in vaccinated gerbils did not show any specific relationship between the titer and level of protection. Lack of significant correlations between antibody titers and the number of worms recovered from the vaccinated gerbils is consistent with the previous report that showed no relationship between endpoint tiers and efficacy of the *Ov*-RAL2 and *Ov*-103 vaccine antigens in the mouse model [19]. Nonetheless, we were able to show that the antibodies could in part be functional and kill i*n vitro B*. *malayi* L3 via the antibody mediated cell cytotoxicity (ADCC); antigen-specific serum in the presence of PEC induced killing of 15–30% L3s. The reduced percentage of L3 killing we observed in this study might have been because only the L3s that were completely immotile with severely granulated and disrupted internal tissues were considered as dead; slow moving larvae with PEC attached to its surface were not included as dead larvae. Hence a stringent scoring system was used to evaluate the killing assay, although overall we observed more L3s were affected by PEC plus antigen specific serum. Secondly, the sera used for *in vitro* killing assay was pooled sera after the second immunization (post V2) as we did not have enough sera of post V3 for testing in these in vitro assays; the antibody titers of sera post V2 was much lower than those post V3, which could have also attributed to the reduced percentage of L3 killing observed.

We did not enumerate or examine the cytokine production from cells present in the circulation or in the lymphoid system at the time of necropsy. These data will be more important and informative if cells are collected at an earlier time points, during the migration of larval parasites which are the likely the major targets of the protective immune responses induced. Nevertheless, we did examine the recovered surviving adult worms with a compound microscope; there was no adherence of cells or formation of granulomas around these adult worms, hence there is no clear direct evidence that vaccination can impact adult worms directly. In previous studies, the mechanism of protection induced by vaccination with irradiated L3 was resolved and shown to be Th2 dependent (IL-4 and/or IL-5) with the essential involvement of IgE and eosinophils against *O*. *volvulus* and *B*. *malayi* in mice [72–74], against *B*. *pahangi* in cats [75], and against *Litomosoides sigmodontis* in mice [76–80]. The lack of immunological reagents specific for gerbils complicates a detailed investigation of comprehensive immunological responses elicited and functional in this animal model. In the past, the measurement of mRNA expression levels of gerbil cytokine genes was used in other experimental system [81], and could be useful in future studies to study the mechanism of protection induced by vaccination with the protective recombinant proteins we studied here.

The protective immunity induced by vaccination with *Bm*-RAL-2 and *Bm*-103 individually or as a fusion protein or when administered concurrently against *B*. *malayi* subcutaneous infection of gerbils supports the findings of Hess et al., 2014 [19] and confirms that these two antigens are important vaccine candidates as they can also control adult worm burden and fecundity of female worms in the gerbil-model, and thus could be used as additional control tools against onchocerciasis and lymphatic filariasis. The fact that these homologous proteins are effective in inducing protective immunity against two filarial nematodes and in two distinct animal models offers a high probability for success at inducing protective immunity in humans as well. Recently, The Onchocerciasis Vaccine for Africa (TOVA) initiative was launched with the aim of developing a prophylactic vaccine for children under the age of five who do not receive ivermectin; *Ov*-103 and *Ov*-RAL-2 are two vaccine candidates that meet the target product profile of TOVA [4], [82].

In summation, although the vaccine efficacy of *Bm*-RAL-2 and *Bm*-103 is reproducible and consistent, vaccination with *Bm*-RAL-2 and *Bm*-103 concurrently or as a fusion protein conferred enhanced levels of protection against a subcutaneous *B*. *malayi* infection in Mongolian gerbils. Specifically, our findings demonstrate that these vaccine candidates when combined together not only induce in gerbils a significant reduction in the *B*. *malayi* adult worm burden but it also reduced the fecundity of surviving female worms. Thus, testing of these vaccine candidates in two animal models, the *O*. *volvulus*- mouse-diffusion chamber model and the *B*. *malayi* -Mongolian gerbil model have proven to be an efficient means to critically confirm the potency of filarial vaccine candidates; vaccine candidates that work in both animal models increase their probability of being protective in humans as well. Further investigation of these vaccine candidates is warranted with work focusing towards product development and manufacturing of a bivalent vaccine. The ultimate goal of this program is the clinical development and testing of a first-generation recombinant filarial vaccine.

## Supporting Information

S1 Dataset Fig S1 A. Sequence alignment of *Bm*-103 and *Ov*-103. Identical amino acid residues are shaded in black, and conservative substitutions are shaded in gray. The sequence identities between the two proteins are added at the end of sequence. Fig S1 B. Phylogenetic analysis of *Bm*-103 (GenBank# XP_001891826.1) with other nematode homologues such as *Ov*-103 (*Onchocerca volvulus*, AAA63412.2); Ll-103 (*Loa loa*, EJD74696.1); *Wb*-103 (*Wuchereria bancrofti*, EJW78103.1); *Ay*-103 (*A*. *ceylanicum*, EPB77163.1); *Na*-103 (*Necator americanus*, XP_013293822.1); *Hc*-103 (*Haemonchus contortus*, KHN78708.1); *Ce*-103 (*Caenorhabditis elegans*, NP_001023598.1), and *Tc*-103 (*Toxocara canis*, KHN78708.1). The data represents neighbor-joining tree representing phylogenetic relationships between nematode 103-like proteins. Fig S2. Immunoelectron microscopic localization of *Ov*-103 and *Bm*-103 in female worms (♀), uterine microfilariae (MF) and L3s (insert shows the granules of the glandular esophagus in L3) of *O*. *volvulus* and *B*. *malayi*, respectively. Abbreviations: hy–hypodermis, cu–cuticle, mu–muscle; bar—500 nm. Fig S3 A. Sequence alignment of *Bm*-RAL-2 and *Ov*-RAL-2. Identical amino acid residues are shaded in black, and conservative substitutions are shaded in gray. The sequence identities between the two proteins are added at the end of sequence. Fig S3 B. Phylogenetic analysis of *Bm*-RAL-2 (GenBank# XP_001900036.1) with other nematode homologues such as *Ov*-RAL-2 (*Onchocerca volvulus*, # P36991.1); *Wb*-RAL-2 (*Wuchereria bancrofti*, AAC17637.1); Ll-RAL-2 (*Loa loa*, AAG09181.1); *Al*-16 (*Ascaris lumbricoides*, ADB45852.1); *Tc*-RAL-2 (*Toxocara canis*, KHN84076.1); *As*-16 (*A*. *suum*, BAC66614.1); *Ac*16 (*Ancylostoma caninum*, ABD98404.1); *As*14 (*A*. *suum*, BAB67769.1); *Ce*-RAL-2 (*Caenorhabditis elegans*, NP_495640.1); Na-SAA-2 (*Necator americanus*, XP_013290850.1); *Ay*-RAL-2 (*A*. *ceylanicum*, EPB72254.1); *Hc*-RAL-2 (*Haemonchus contortus*, CDJ91573.1), and *Ad*-RAL-2 (*A*. *duodenale*, KIH68079.1). The data represents neighbor-joining tree representing phylogenetic relationships between nematode RAL-2-like proteins. Fig S4. Immunoelectron microscopic localization of *Ov*-RAL-2 and *Bm*-RAL-2 in female worms (♀), uterine microfilariae (MF) and L3s (insert shows the granules of the glandular esophagus in L3) of *O*. *volvulus* and *B*. *malayi*, respectively. Abbreviations: hy–hypodermis, cu–cuticle; bar—500 nm. Fig S5 Sequence alignment of *Bm*-103 –*Bm*-RAL-2 and *Ov*-103 –*Ov-*RAL-2 fusion proteins. The amino acid sequence corresponding to 103 and RAL-2 are squared in black and red, respectively. The linker is squared in blue. Identical amino acid residues are shaded in black and conservative substitutions are shaded in gray. The sequence identities and similarities between the two fusion proteins are added at the end of sequence.
(PPTX)

S2 Dataset Fig S1. Experiment 1; Female: male ratio in gerbils vaccinated with *Bm*-RAL-2 (42 dpi). Each dot represents the total number of adult worms recovered from an individual animal. The lines represent mean with standard deviation. *P* ≤ 0.05 denotes a statistically significant difference in total worm recovery (percent reduction) between vaccinated and alum control group, Mann–Whitney U Test, GraphPad Prism 6. Fig S2. Experiment 1; Female: male ratio in gerbils vaccinated with *Bm*-103 (42 dpi). Each dot represents the total number of adult worms recovered from an individual animal. The lines represent mean with standard deviation. *P* ≤ 0.05 denotes a statistically significant difference in total worm recovery (percent reduction) between vaccinated and alum control group, Mann–Whitney U Test, GraphPad Prism 6. Fig S3. Experiment 3; Female: male ratio in gerbils vaccinated with *Bm*-RAL-2 (120 dpi) Each dot represents the total number of adult worms recovered from an individual animal. The lines represent mean with standard deviation. *P* ≤ 0.05 denotes a statistically significant difference in total worm recovery (percent reduction) between vaccinated and alum control group, Mann–Whitney U Test, GraphPad Prism 6. Fig S4. Experiment 4; Female: male ratio in gerbils vaccinated with *Bm*-103 vaccination (120 dpi). Each dot represents the total number of adult worms recovered from an individual animal. The lines represent mean with standard deviation. *P* ≤ 0.05 denotes a statistically significant difference in total worm recovery (percent reduction) between vaccinated and alum control group, Mann–Whitney U Test, GraphPad Prism 6. Fig S5. Experiment 5; Female: male ratio in gerbils vaccinated with *Bm*-RAL-2 and *Bm*-103 (150 dpi). Each dot represents the total number of adult worms recovered from an individual animal. The lines represent mean with standard deviation. *P* ≤ 0.05 denotes a statistically significant difference in total worm recovery (percent reduction) between vaccinated and alum control group, Mann–Whitney U Test, GraphPad Prism 6. Fig S6. Experiment 6; Female: male ratio in gerbils vaccinated with *Bm*-RAL-2 and *Bm*-103 concurrently (90 dpi). The lines represent mean with standard deviation. *P* ≤ 0.05 denotes a statistically significant difference in total worm recovery (percent reduction) between vaccinated and alum control group, Mann–Whitney U Test, GraphPad Prism 6. Fig S7. Experiment 7; Female: male ratio in gerbils vaccinated with *Bm*-RAL-2 and *Bm*-103 as a fusion antigen vaccination (90 dpi). The lines represent mean with standard deviation. *P* ≤ 0.05 denotes a statistically significant difference in total worm recovery (percent reduction) between vaccinated and alum control group, Mann–Whitney U Test, GraphPad Prism 6. Fig S8. Experiment 8; Female: male ratio in gerbils vaccinated with *Bm*-RAL-2 and *Bm*-103 as fusion protein and concurrently (90 dpi). The lines represent mean with standard deviation. *P* ≤ 0.05 denotes a statistically significant difference in total worm recovery (percent reduction) between vaccinated and alum control group, Mann–Whitney U Test, GraphPad Prism 6. Fig S9. Experiment 9; Female: male ratio in gerbils vaccinated with *Bm*-RAL-2 and *Bm*-103 as fusion protein and concurrently (150 dpi). The lines represent mean with standard deviation. *P* ≤ 0.05 denotes a statistically significant difference in total worm recovery (percent reduction) between vaccinated and alum control group, Mann–Whitney U Test, GraphPad Prism 6.
(PPTX)

S3 Dataset Fig S1. Experiment 5, microfilariae levels in alum controls and gerbils vaccinated with *Bm*-103 and *Bm*-RAL-2 150 dpi. Statistical significance was determined by Mann–Whitney U Test using GraphPad Prism version 6, asterisks denotes a significant difference between alum controls and vaccinated group, *P* ≤ 0.05. The line represents mean with standard deviation. Fig S2. Experiment 9, microfilariae levels in alum controls and gerbils vaccinated with *Bm*-103 and *Bm*-RAL-2 fusion and concurrent vaccines 150 dpi. Statistical significance was determined by Mann–Whitney U Test using GraphPad Prism version 6, asterisks denotes a significant difference between alum controls and vaccinated group, *P* ≤ 0.05. The line represents mean with standard deviation. Fig S3. A survey of microfilariae (MF) levels in gerbils infected with *B*. *malayi* over a period of 3 years. Gerbils were infected with 150 infective *B*. *malayi* L3 larvae and gerbils were bled periodically and MF levels in 20 μl of gerbil blood were enumerated under a light microscopy.
(PPTX)

S1 Table Number of worms recovered from the lymphatics and from the heart & lungs of gerbils vaccinated with *Bm*-RAL-2 and *Bm*-103 individually, concurrently or as a fusion protein. (DOCX)

S1 Video Control assay, L3 larvae cultured in the presence of serum from gerbils injected with alum and peritoneal exudate cells (PEC). (AVI)

S2 Video *In vitro* killing of L3 larvae in the presence of anti-*Bm*-103 serum and peritoneal exudate cells (PEC). (AVI)
