# Supplementary material for: Vaccination of Gerbils with Bm-103 and Bm-RAL-2 Concurrently or as a Fusion Protein Confers Consistent and Improved Protection against Brugia malayi Infection
Source: PLoS Negl Trop Dis. 2016 Apr 5;10(4):e0004586. doi: 10.1371/journal.pntd.0004586 (PMC4821550; doi:10.1371/journal.pntd.0004586)
Supplement: S1 Dataset — Fig S1 A. Sequence alignment of Bm-103 and Ov-103. Identical amino acid residues are shaded in black, and conservative substitutions are shaded in gray. The sequence identities between the two proteins are added at the end of sequence. Fig S1 B. Phylogenetic analysis of Bm-103 (GenBank# XP_001891826.1) with other nematode homologues such as Ov-103 (Onchocerca volvulus, AAA63412.2); Ll-103 (Loa loa, EJD74696.1); Wb-103 (Wuchereria bancrofti, EJW78103.1); Ay-103 (A. ceylanicum, EPB77163.1); Na-103 (Necator americanus, XP_013293822.1); Hc-103 (Haemonchus contortus, KHN78708.1); Ce-103 (Caenorhabditis elegans, NP_001023598.1), and Tc-103 (Toxocara canis, KHN78708.1). The data represents neighbor-joining tree representing phylogenetic relationships between nematode 103-like proteins. Fig S2. Immunoelectron microscopic localization of Ov-103 and Bm-103 in female worms (♀), uterine microfilariae (MF) and L3s (insert shows the granules of the glandular esophagus in L3) of O. volvulus and B. malayi, respectively. Abbreviations: hy–hypodermis, cu–cuticle, mu–muscle; bar—500 nm. Fig S3 A. Sequence alignment of Bm-RAL-2 and Ov-RAL-2. Identical amino acid residues are shaded in black, and conservative substitutions are shaded in gray. The sequence identities between the two proteins are added at the end of sequence. Fig S3 B. Phylogenetic analysis of Bm-RAL-2 (GenBank# XP_001900036.1) with other nematode homologues such as Ov-RAL-2 (Onchocerca volvulus, # P36991.1); Wb-RAL-2 (Wuchereria bancrofti, AAC17637.1); Ll-RAL-2 (Loa loa, AAG09181.1); Al-16 (Ascaris lumbricoides, ADB45852.1); Tc-RAL-2 (Toxocara canis, KHN84076.1); As-16 (A. suum, BAC66614.1); Ac16 (Ancylostoma caninum, ABD98404.1); As14 (A. suum, BAB67769.1); Ce-RAL-2 (Caenorhabditis elegans, NP_495640.1); Na-SAA-2 (Necator americanus, XP_013290850.1); Ay-RAL-2 (A. ceylanicum, EPB72254.1); Hc-RAL-2 (Haemonchus contortus, CDJ91573.1), and Ad-RAL-2 (A. duodenale, KIH68079.1). The data represents neighbor-joining tree repres [file pntd.0004586.s001.pptx]

## Slide 1
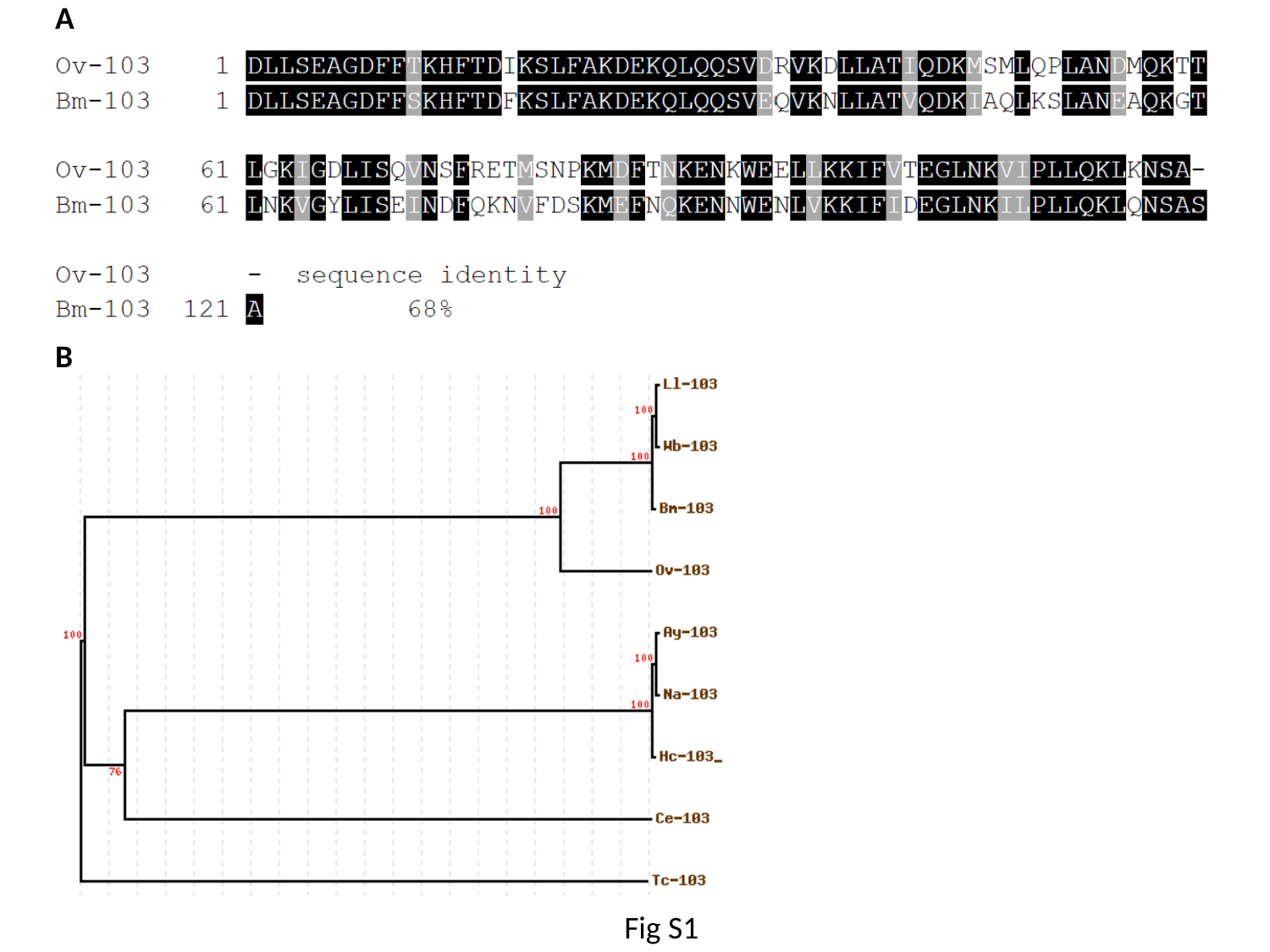

A
B
Fig S1

## Slide 2
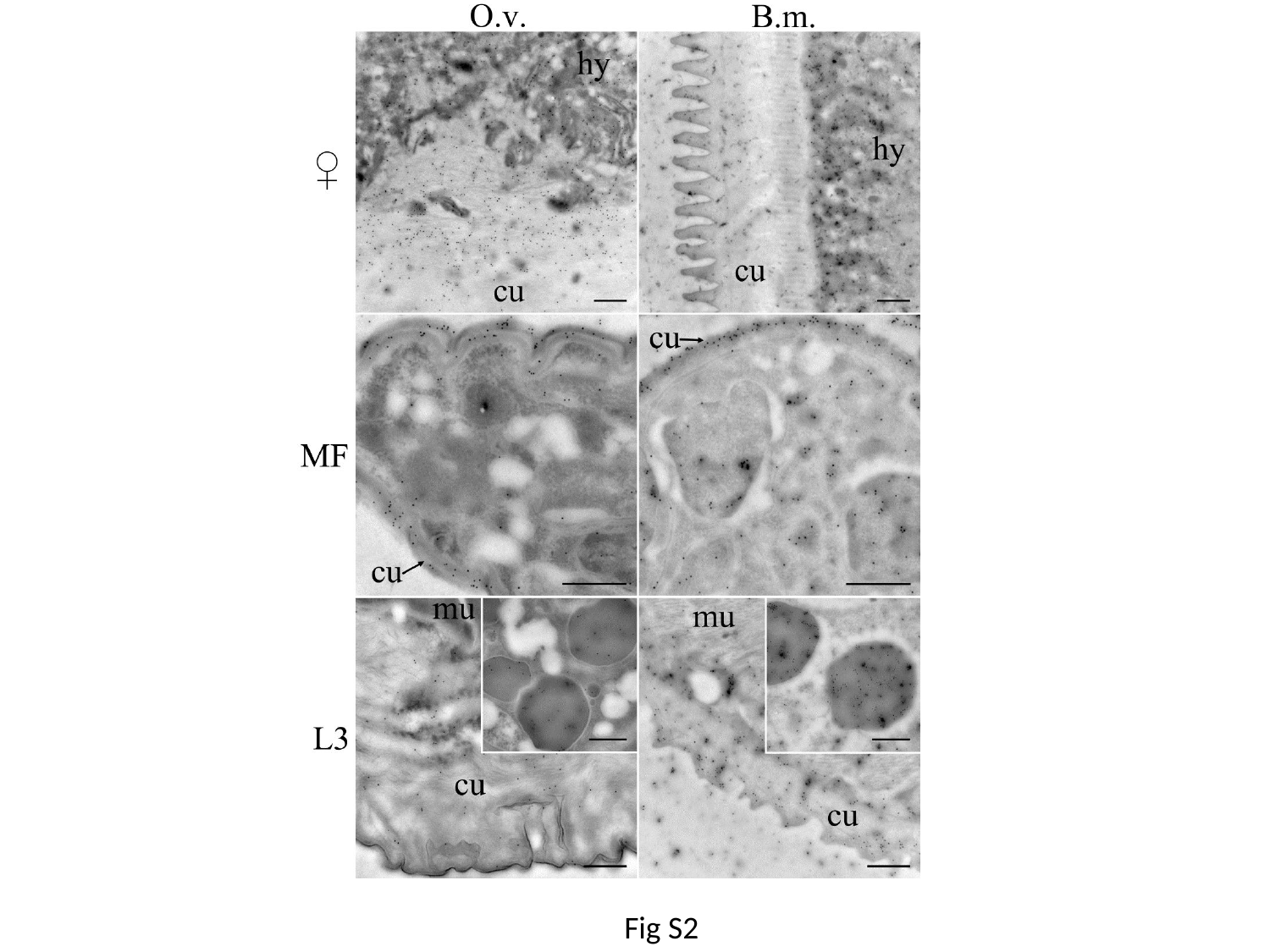

Fig S2

## Slide 3
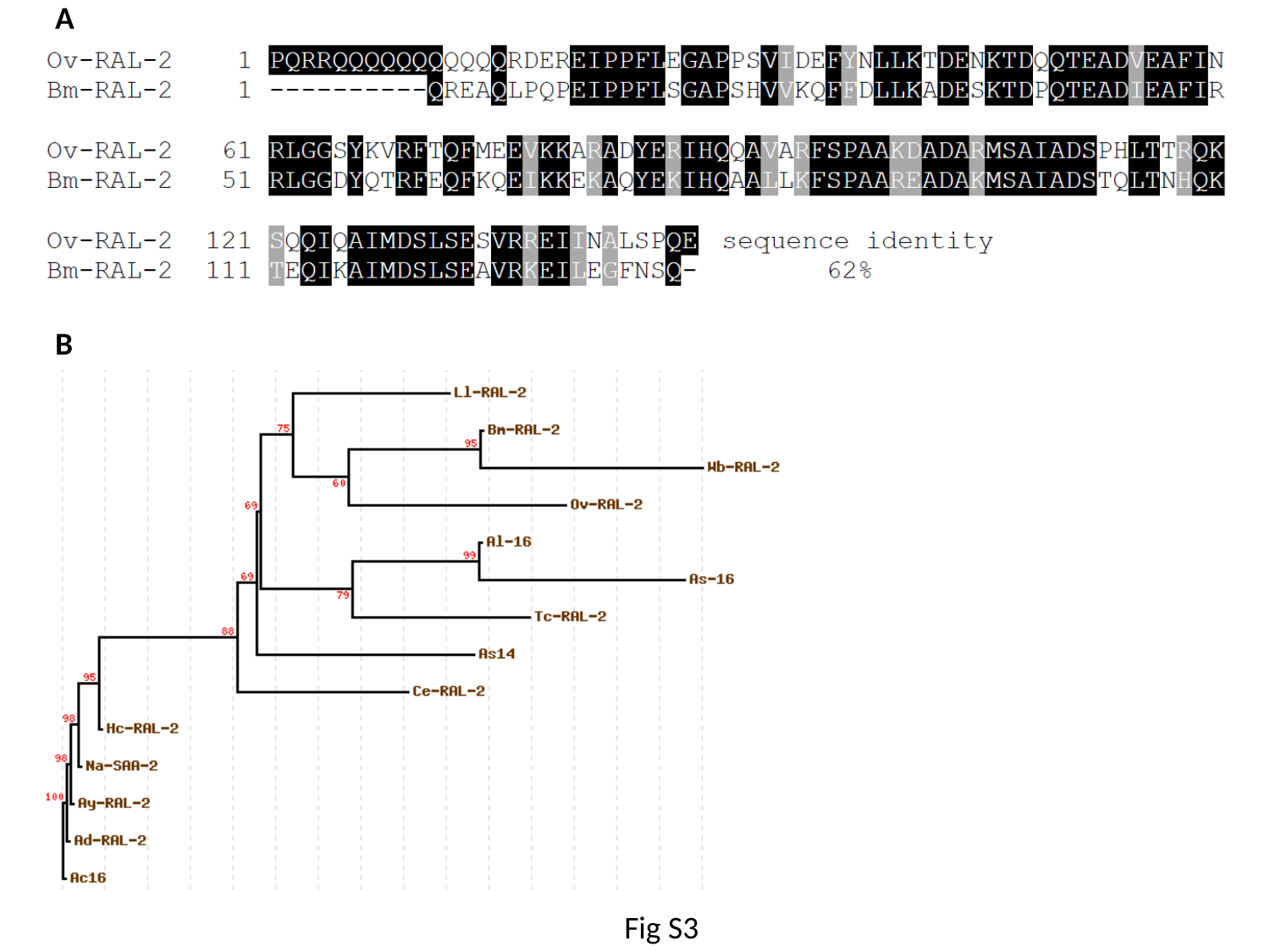

A
B
Fig S3

## Slide 4
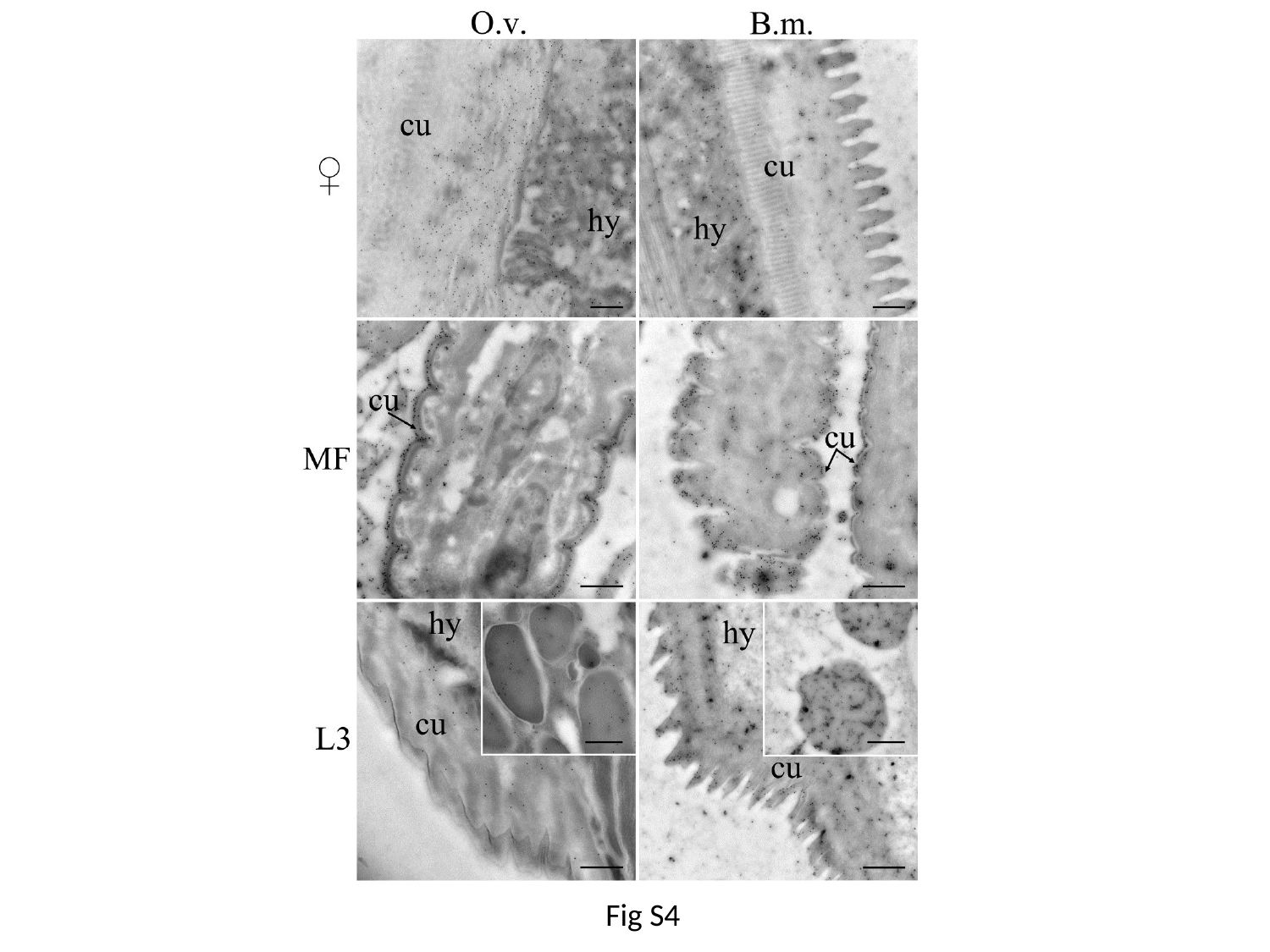

Fig S4

## Slide 5
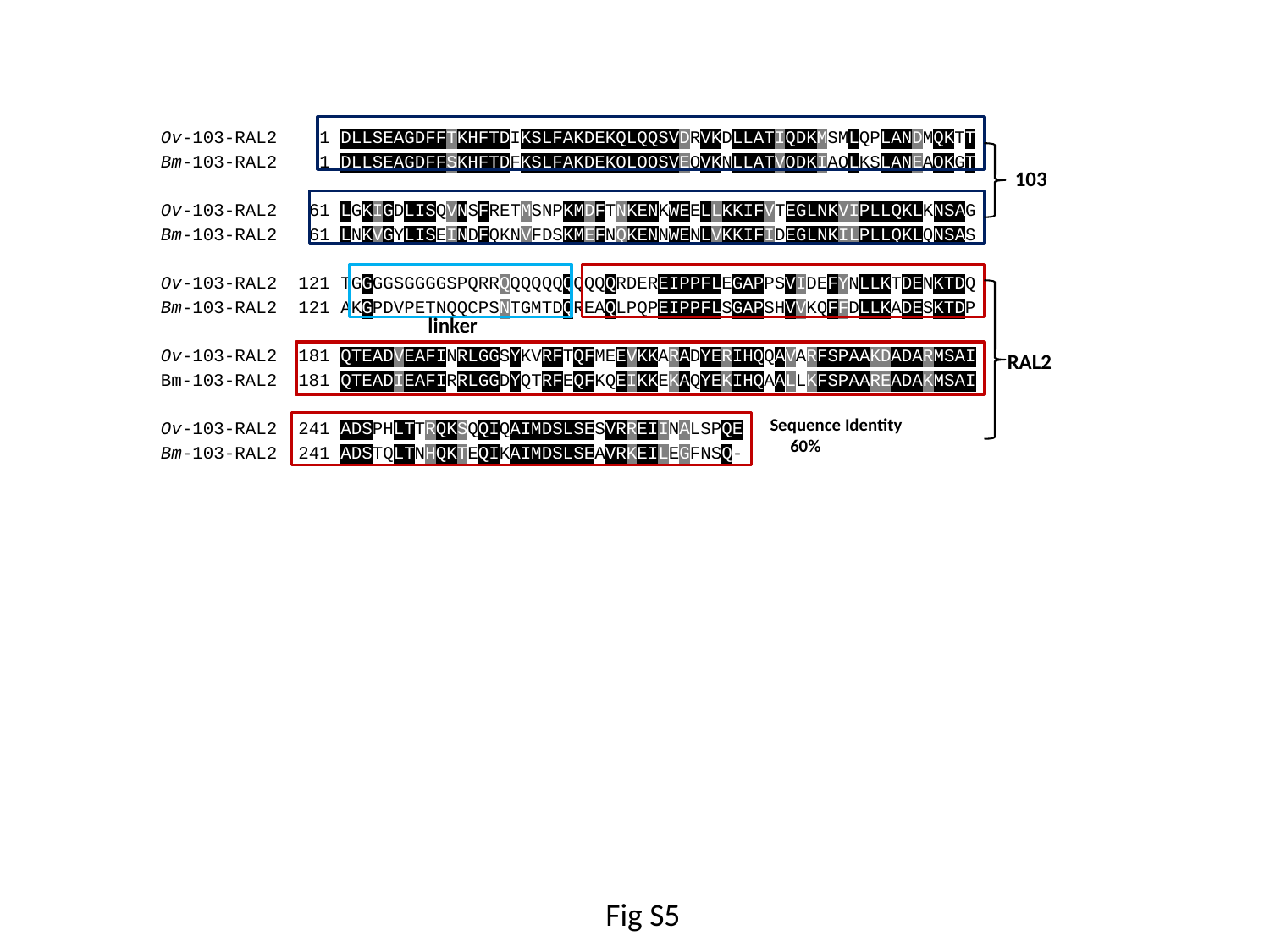

Ov-103-RAL2 1 DLLSEAGDFFTKHFTDIKSLFAKDEKQLQQSVDRVKDLLATIQDKMSMLQPLANDMQKTT
Bm-103-RAL2 1 DLLSEAGDFFSKHFTDFKSLFAKDEKQLQQSVEQVKNLLATVQDKIAQLKSLANEAQKGT
Ov-103-RAL2 61 LGKIGDLISQVNSFRETMSNPKMDFTNKENKWEELLKKIFVTEGLNKVIPLLQKLKNSAG
Bm-103-RAL2 61 LNKVGYLISEINDFQKNVFDSKMEFNQKENNWENLVKKIFIDEGLNKILPLLQKLQNSAS
Ov-103-RAL2 121 TGGGGSGGGGSPQRRQQQQQQQQQQQRDEREIPPFLEGAPPSVIDEFYNLLKTDENKTDQ
Bm-103-RAL2 121 AKGPDVPETNQQCPSNTGMTDQREAQLPQPEIPPFLSGAPSHVVKQFFDLLKADESKTDP
Ov-103-RAL2 181 QTEADVEAFINRLGGSYKVRFTQFMEEVKKARADYERIHQQAVARFSPAAKDADARMSAI
Bm-103-RAL2 181 QTEADIEAFIRRLGGDYQTRFEQFKQEIKKEKAQYEKIHQAALLKFSPAAREADAKMSAI
Ov-103-RAL2 241 ADSPHLTTRQKSQQIQAIMDSLSESVRREIINALSPQE
Bm-103-RAL2 241 ADSTQLTNHQKTEQIKAIMDSLSEAVRKEILEGFNSQ-
103
linker
RAL2
Sequence Identity
 60%
Fig S5
