# Supplementary material for: Vaccination of Gerbils with Bm-103 and Bm-RAL-2 Concurrently or as a Fusion Protein Confers Consistent and Improved Protection against Brugia malayi Infection
Source: PLoS Negl Trop Dis. 2016 Apr 5;10(4):e0004586. doi: 10.1371/journal.pntd.0004586 (PMC4821550; doi:10.1371/journal.pntd.0004586)
Supplement: S2 Dataset — Fig S1. Experiment 1; Female: male ratio in gerbils vaccinated with Bm-RAL-2 (42 dpi). Each dot represents the total number of adult worms recovered from an individual animal. The lines represent mean with standard deviation. P ≤ 0.05 denotes a statistically significant difference in total worm recovery (percent reduction) between vaccinated and alum control group, Mann–Whitney U Test, GraphPad Prism 6. Fig S2. Experiment 1; Female: male ratio in gerbils vaccinated with Bm-103 (42 dpi). Each dot represents the total number of adult worms recovered from an individual animal. The lines represent mean with standard deviation. P ≤ 0.05 denotes a statistically significant difference in total worm recovery (percent reduction) between vaccinated and alum control group, Mann–Whitney U Test, GraphPad Prism 6. Fig S3. Experiment 3; Female: male ratio in gerbils vaccinated with Bm-RAL-2 (120 dpi) Each dot represents the total number of adult worms recovered from an individual animal. The lines represent mean with standard deviation. P ≤ 0.05 denotes a statistically significant difference in total worm recovery (percent reduction) between vaccinated and alum control group, Mann–Whitney U Test, GraphPad Prism 6. Fig S4. Experiment 4; Female: male ratio in gerbils vaccinated with Bm-103 vaccination (120 dpi). Each dot represents the total number of adult worms recovered from an individual animal. The lines represent mean with standard deviation. P ≤ 0.05 denotes a statistically significant difference in total worm recovery (percent reduction) between vaccinated and alum control group, Mann–Whitney U Test, GraphPad Prism 6. Fig S5. Experiment 5; Female: male ratio in gerbils vaccinated with Bm-RAL-2 and Bm-103 (150 dpi). Each dot represents the total number of adult worms recovered from an individual animal. The lines represent mean with standard deviation. P ≤ 0.05 denotes a statistically significant difference in total worm recovery (percent reduction) between vaccinated and alu [file pntd.0004586.s002.pptx]

## Slide 1
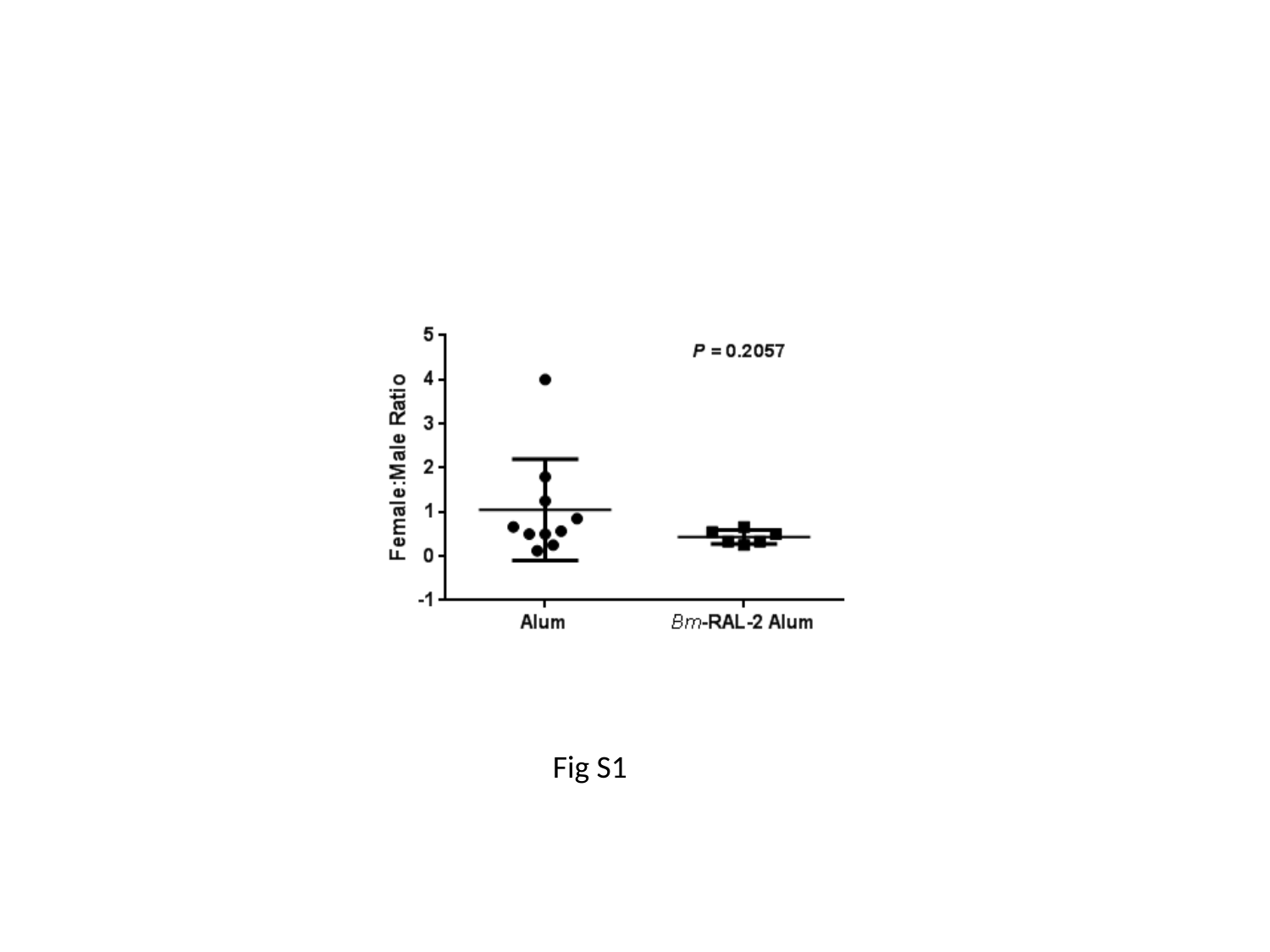

Fig S1

## Slide 2
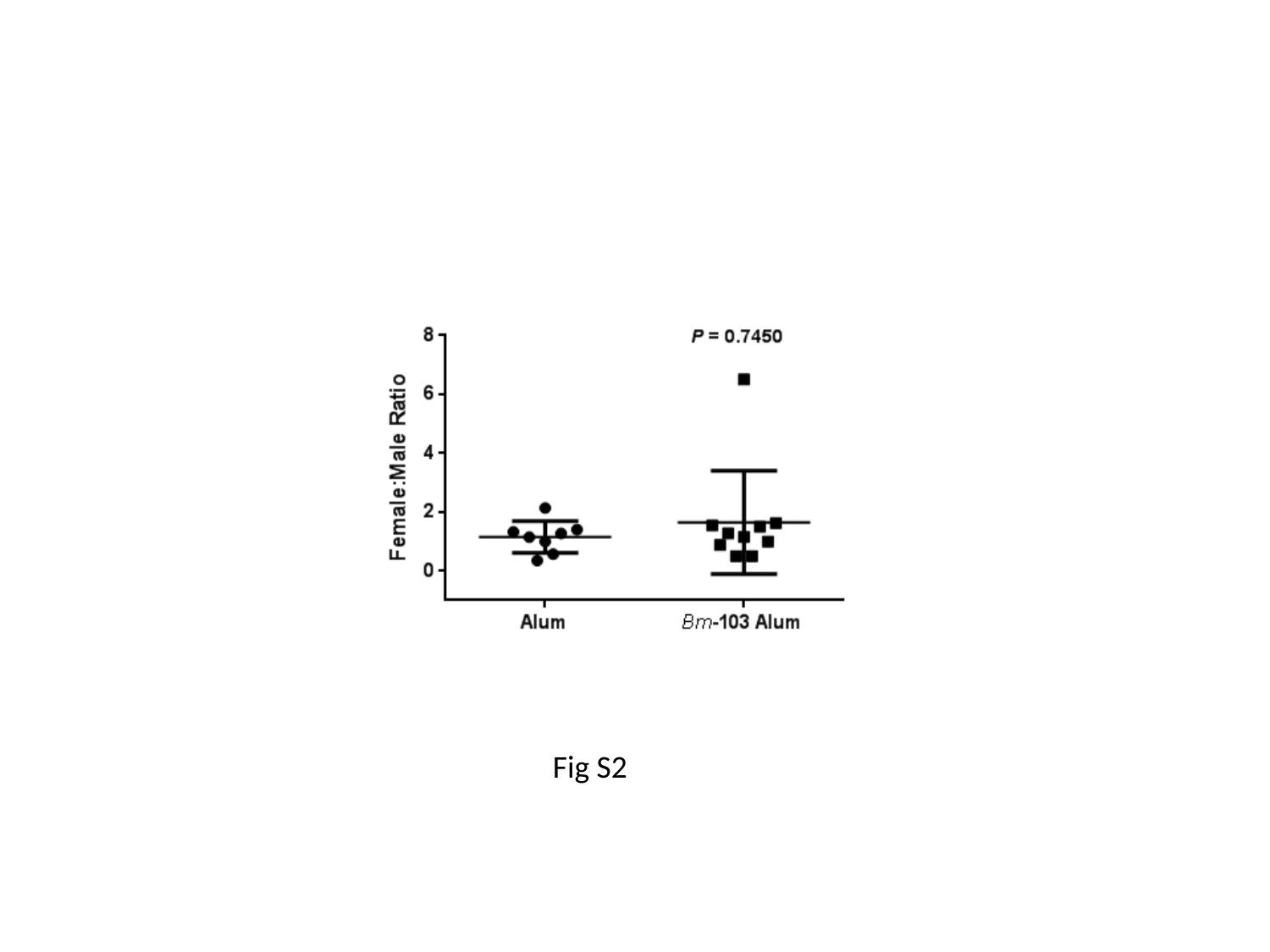

Fig S2

## Slide 3
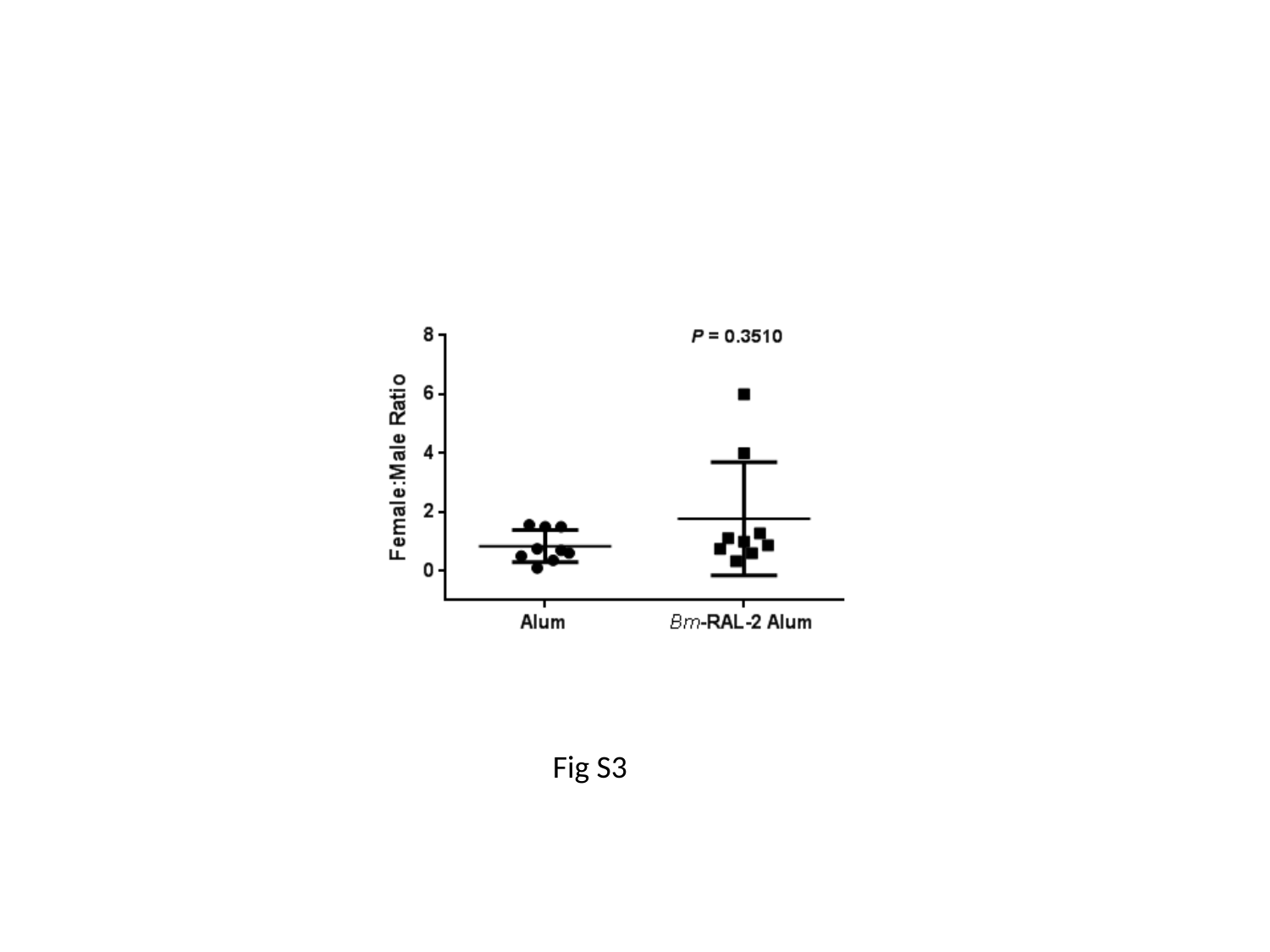

Fig S3

## Slide 4
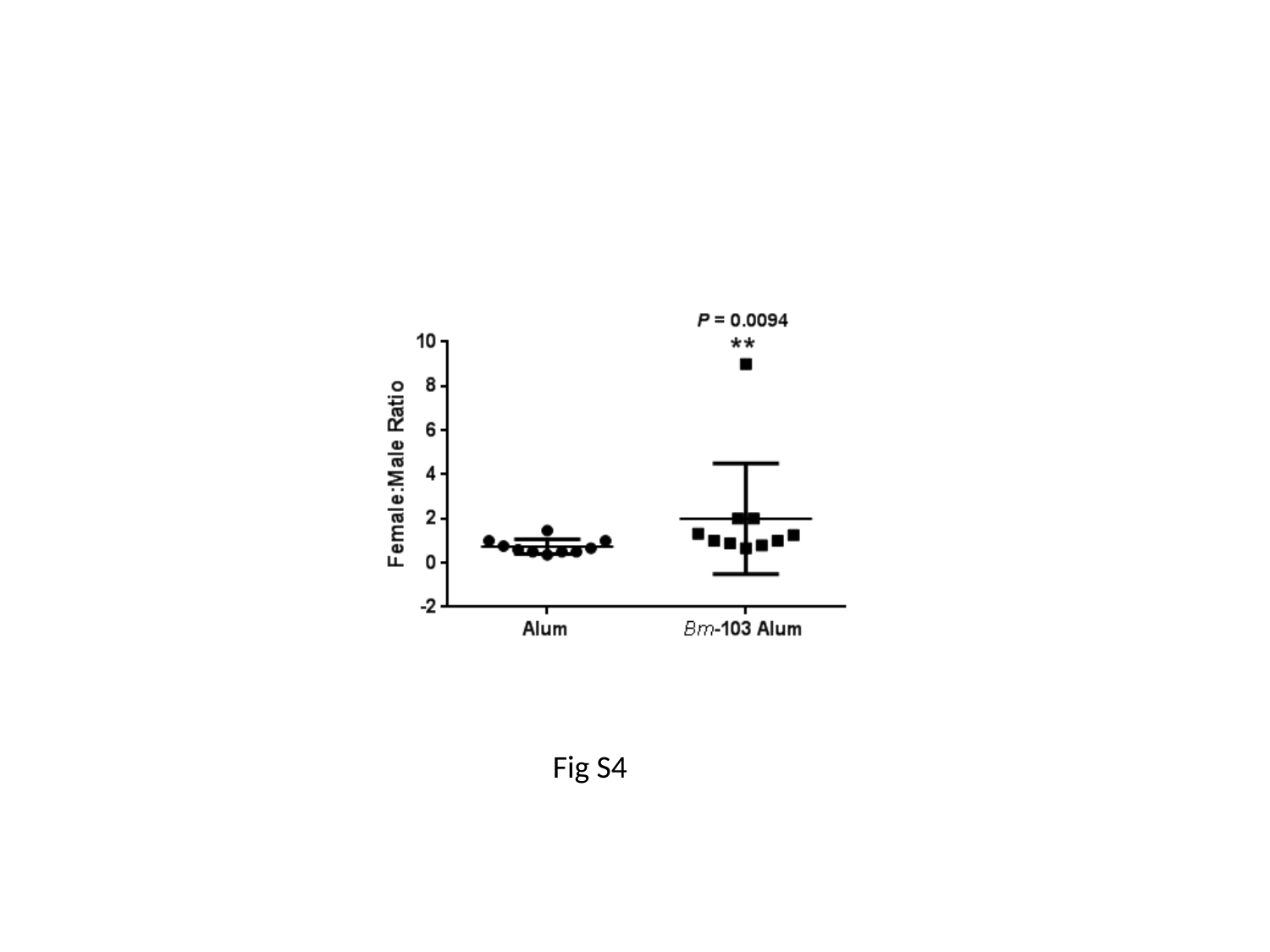

Fig S4

## Slide 5
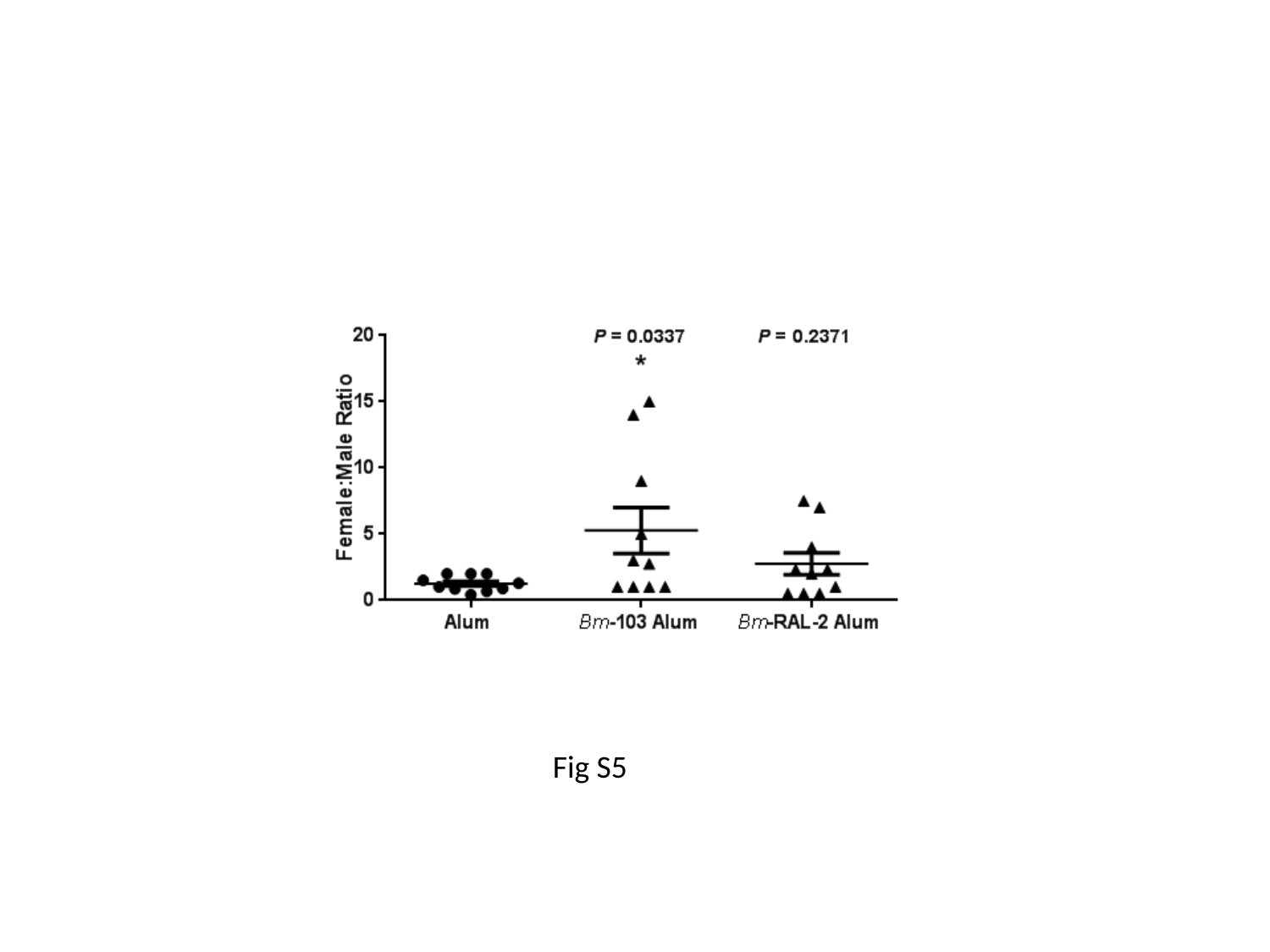

Fig S5

## Slide 6
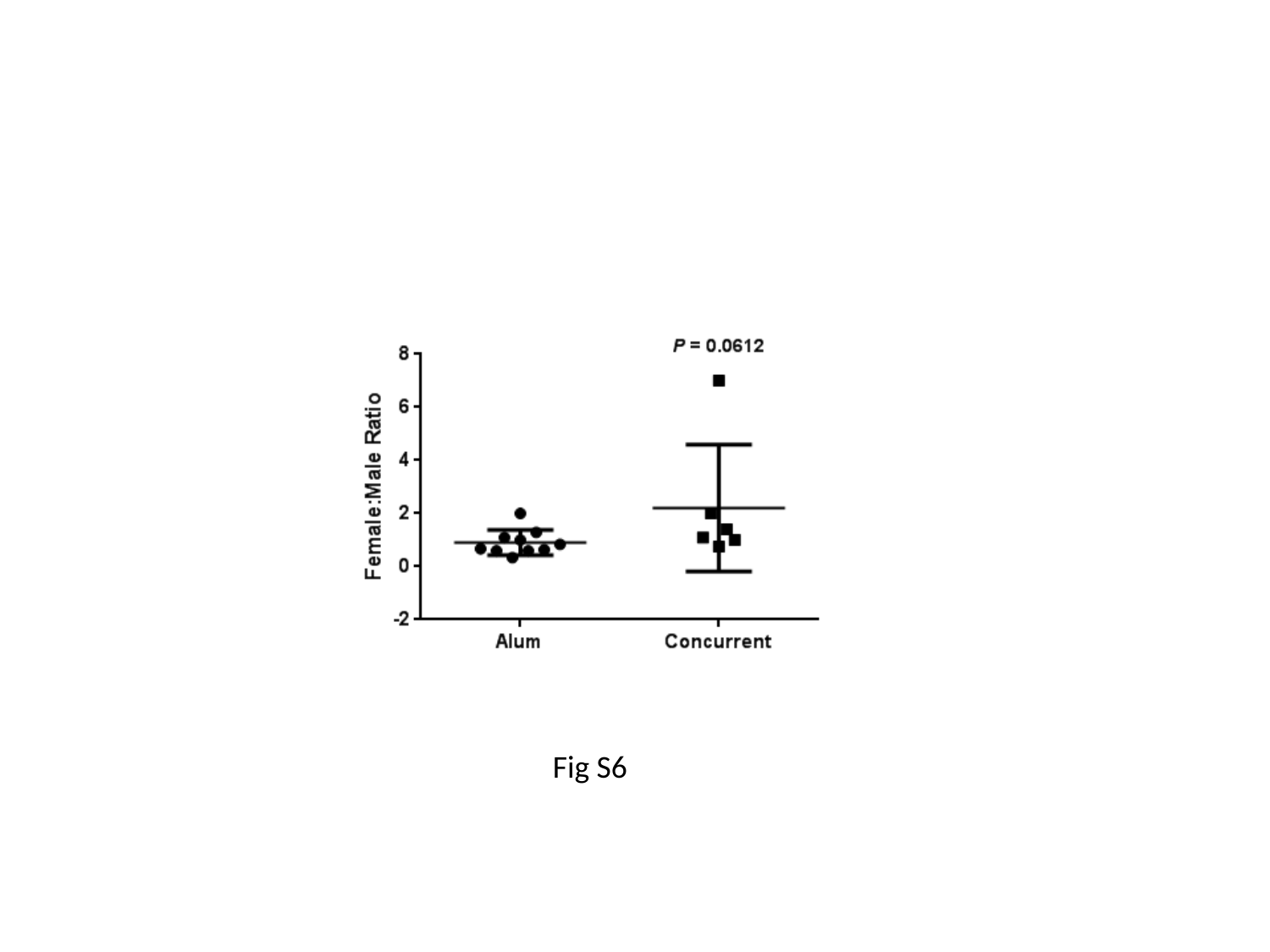

Fig S6

## Slide 7
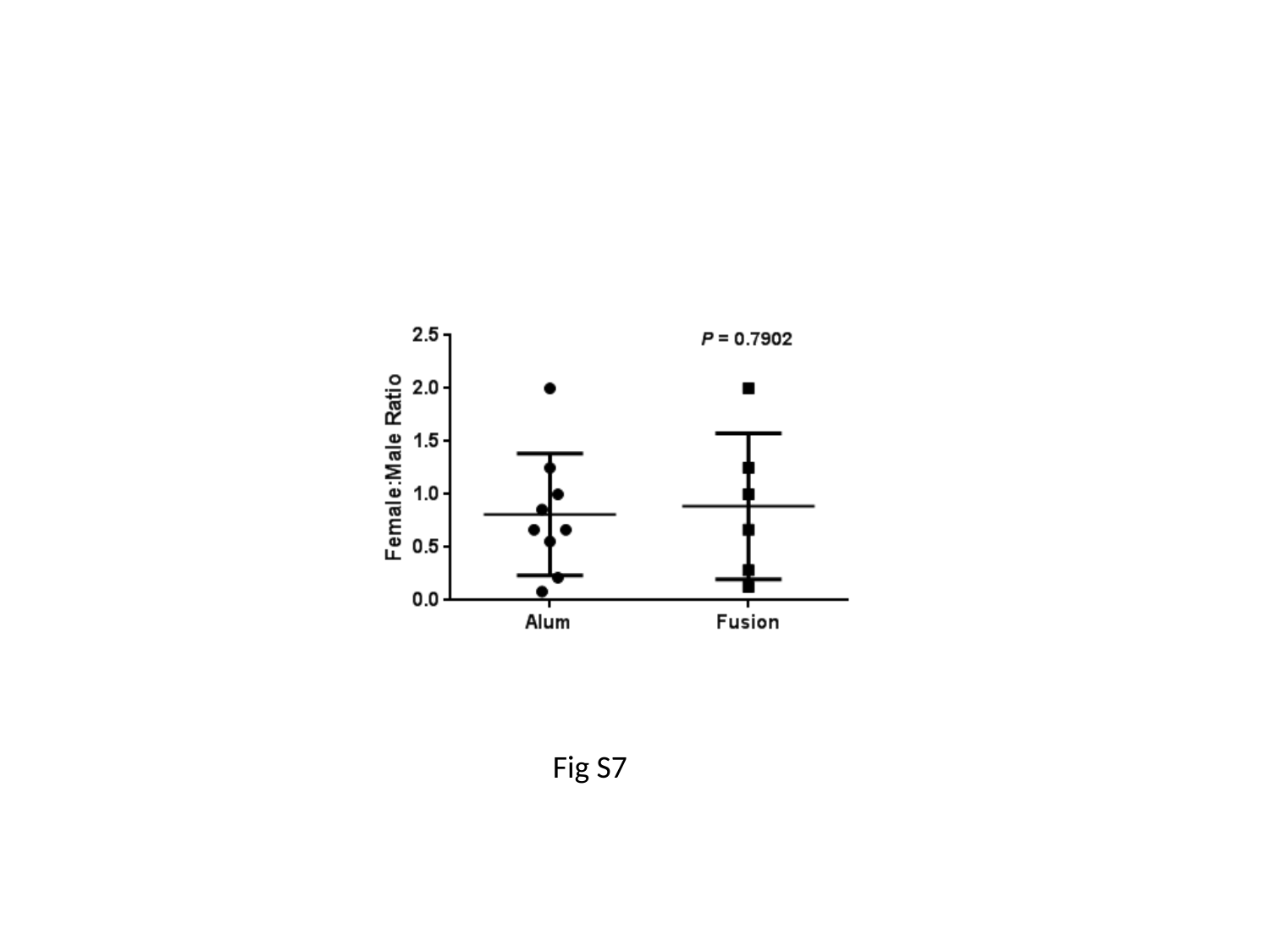

Fig S7

## Slide 8
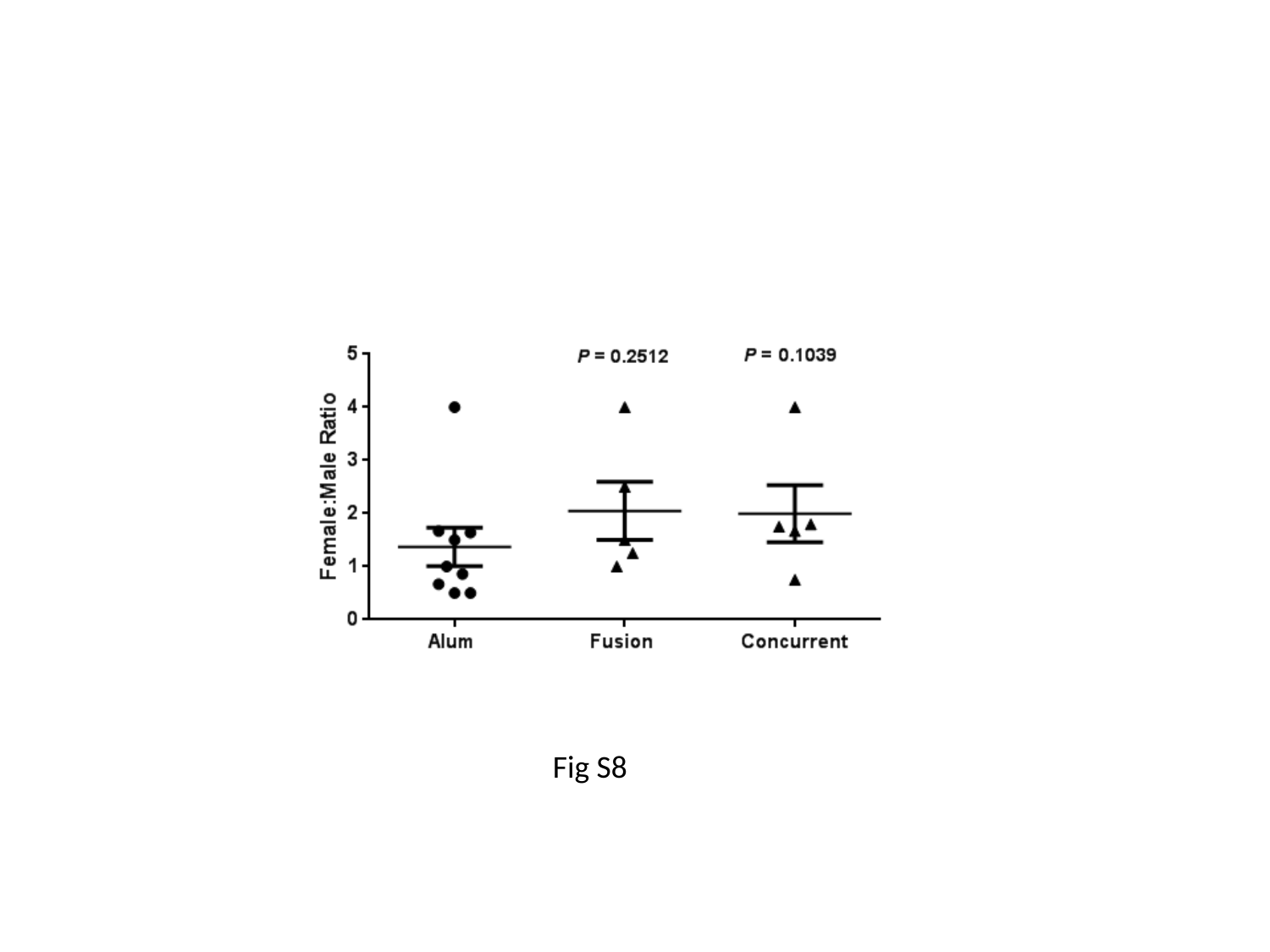

Fig S8

## Slide 9
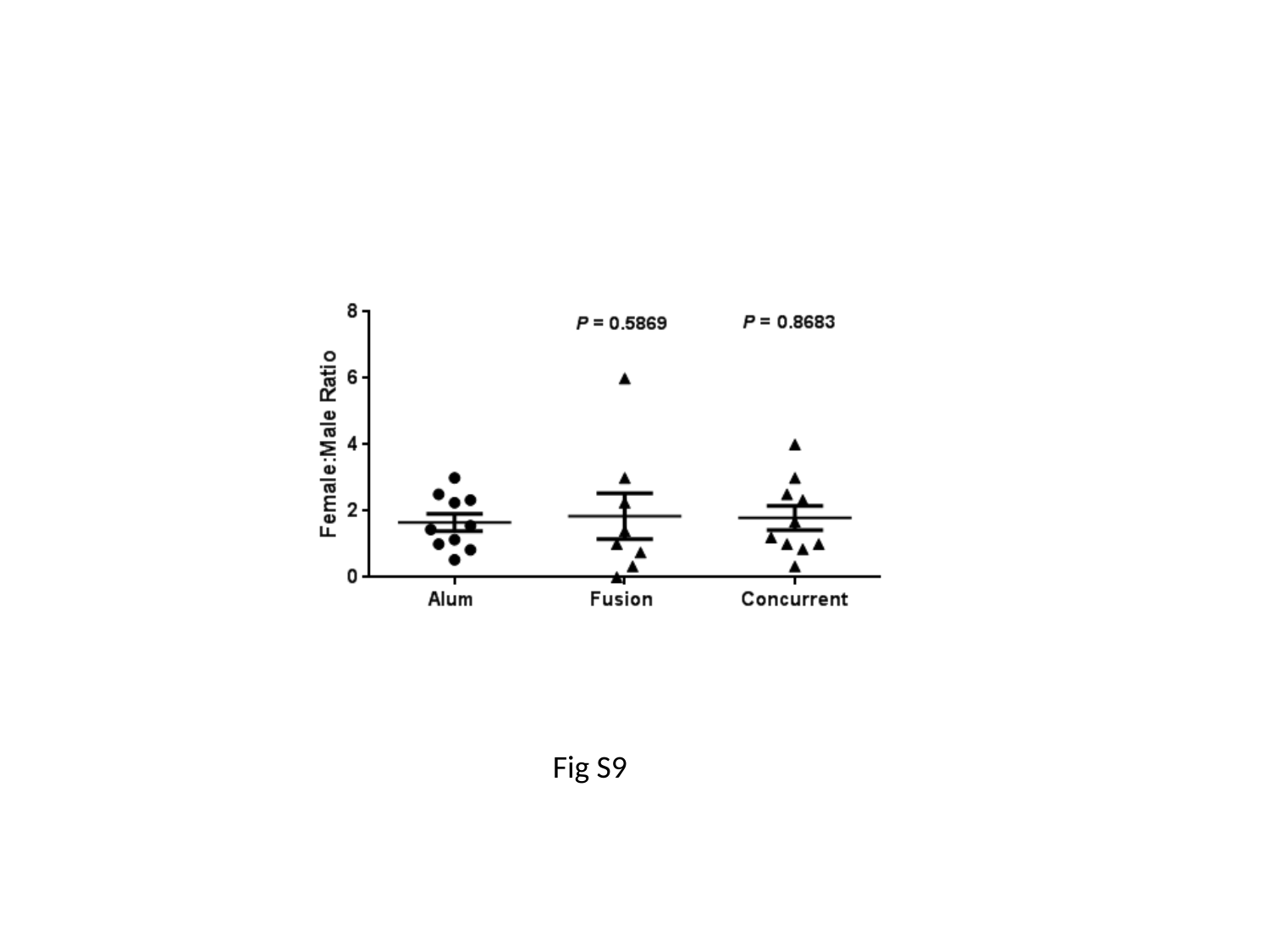

Fig S9
