# Supplementary figures and images for: Vaccination of Gerbils with Bm-103 and Bm-RAL-2 Concurrently or as a Fusion Protein Confers Consistent and Improved Protection against Brugia malayi Infection
Source: PLoS Negl Trop Dis. 2016 Apr 5;10(4):e0004586. doi: 10.1371/journal.pntd.0004586 (PMC4821550; doi:10.1371/journal.pntd.0004586)

## Slide 1
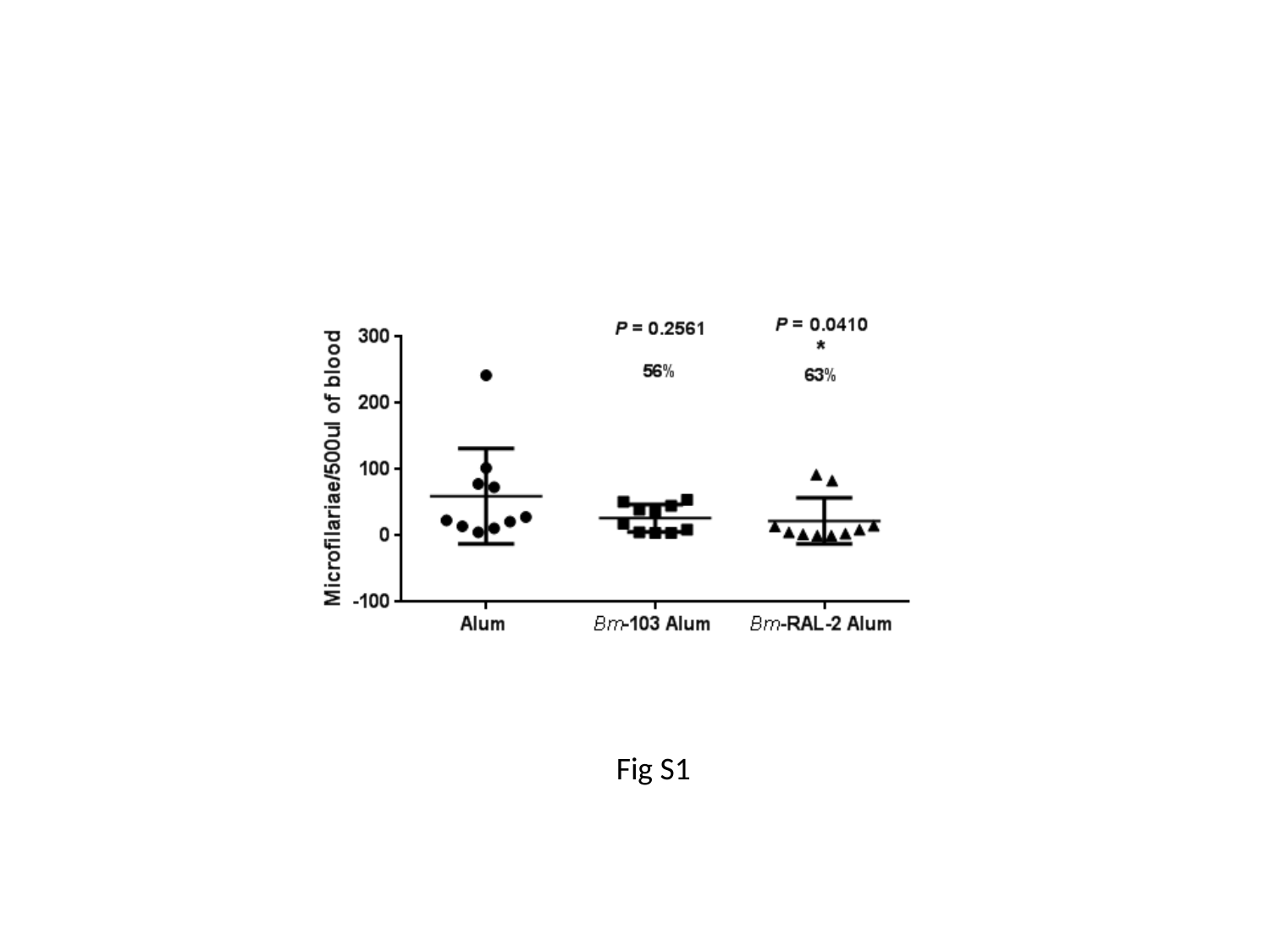

Fig S1

## Slide 2
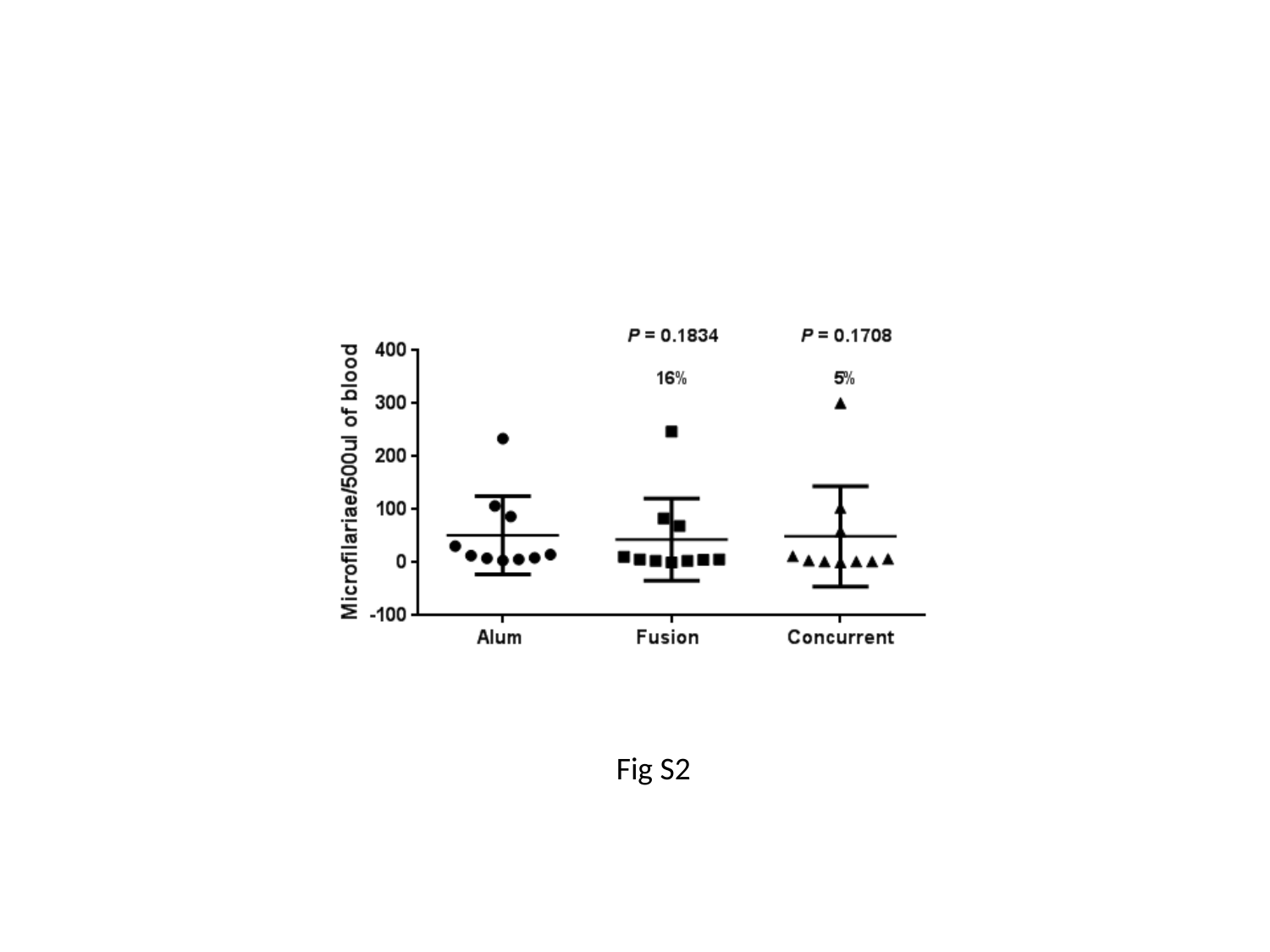

Fig S2

## Slide 3
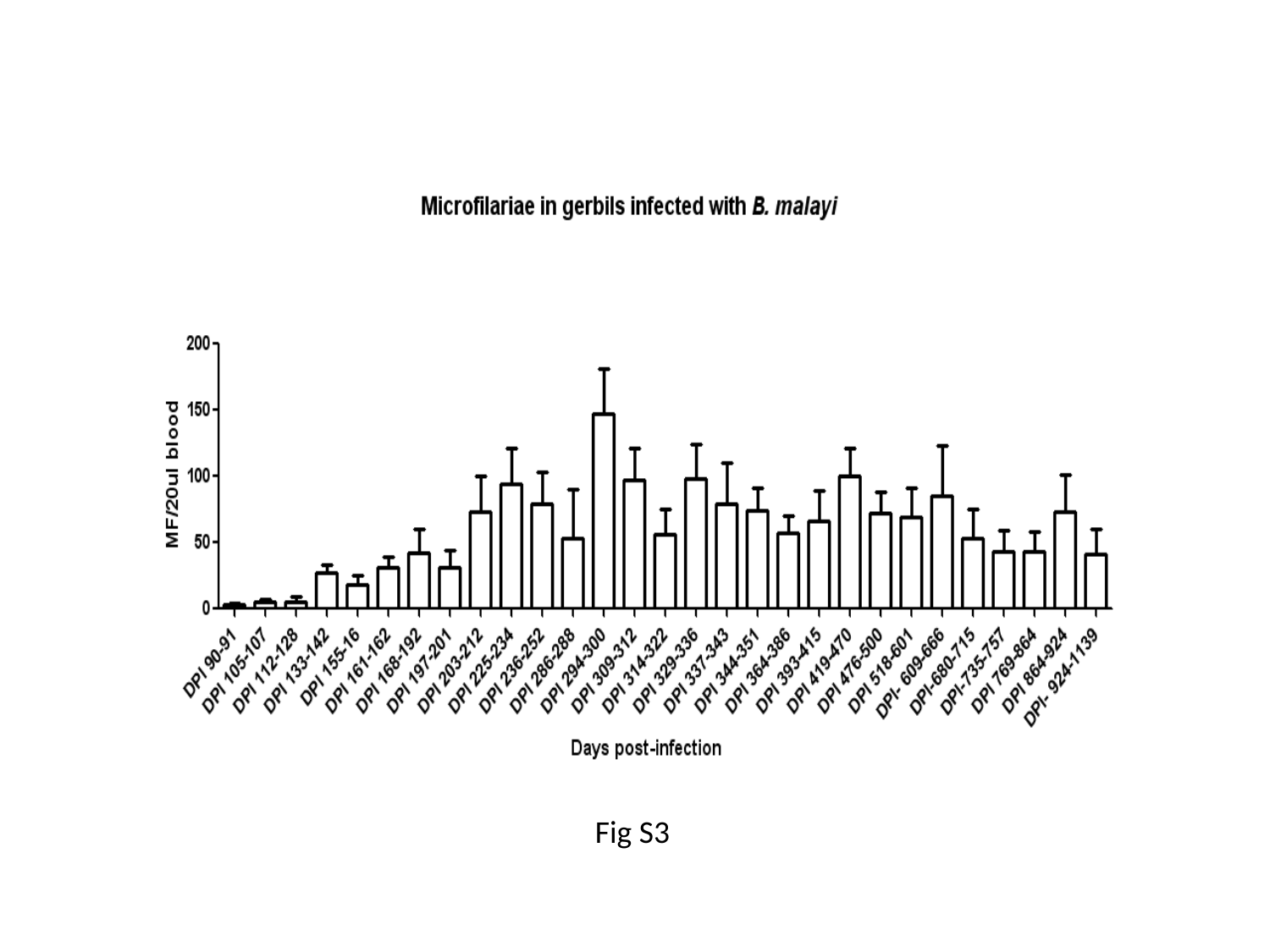

Fig S3

Supplement: S3 Dataset — Fig S1. Experiment 5, microfilariae levels in alum controls and gerbils vaccinated with Bm-103 and Bm-RAL-2 150 dpi. Statistical significance was determined by Mann–Whitney U Test using GraphPad Prism version 6, asterisks denotes a significant difference between alum controls and vaccinated group, P ≤ 0.05. The line represents mean with standard deviation. Fig S2. Experiment 9, microfilariae levels in alum controls and gerbils vaccinated with Bm-103 and Bm-RAL-2 fusion and concurrent vaccines 150 dpi. Statistical significance was determined by Mann–Whitney U Test using GraphPad Prism version 6, asterisks denotes a significant difference between alum controls and vaccinated group, P ≤ 0.05. The line represents mean with standard deviation. Fig S3. A survey of microfilariae (MF) levels in gerbils infected with B. malayi over a period of 3 years. Gerbils were infected with 150 infective B. malayi L3 larvae and gerbils were bled periodically and MF levels in 20 μl of gerbil blood were enumerated under a light microscopy. (PPTX) [file pntd.0004586.s003.pptx]
