# Supplementary material for: Vaccination of Gerbils with Bm-103 and Bm-RAL-2 Concurrently or as a Fusion Protein Confers Consistent and Improved Protection against Brugia malayi Infection
Source: PLoS Negl Trop Dis. 2016 Apr 5;10(4):e0004586. doi: 10.1371/journal.pntd.0004586 (PMC4821550; doi:10.1371/journal.pntd.0004586)
Supplement: S1 Table — (DOCX) [file pntd.0004586.s004.docx]

Supplementary Table 1. Number of worms recovered from the lymphatics and from the heart & lungs of gerbils vaccinated with *Bm*-RAL-2 and *Bm*-103 individually, concurrently or as a fusion protein.

|  |  | Alum control group | | Vaccinated group | |
| --- | --- | --- | --- | --- | --- |
| Exp. No. | Vaccine | Number of worms in the Lymphatics Mean ± SD | Number of worms in the Heart & Lungs  Mean ± SD | Number of worms in the Lymphatics Mean ± SD | Number of worms in the Heart & Lungs  Mean ± SD |
| 1 | *Bm*-RAL-2 (42 dpi) | 7.7 ± 7.0 | 4.3 ± 5.4 | 6.1 ± 4.3 | 0.6 ± 1.2 |
| 2 | *Bm*-103 (42 dpi) | 11.9 ± 7.9 | 8.5 ± 6.4 | 8.0 ± 7.8 | 4.7 ± 4.1 |
| 3 | *Bm*-RAL-2 (120 dpi) | 6.4 ± 7.3 | 6.0 ± 6.2 | 5.7 ± 5.3 | 4.0 ± 5.7 |
| 4 | *Bm*-103 (120 dpi) | 13.0 ± 10.7 | 5.8 ± 6.2 | 9.0 ± 6.5 | 3.8 ± 4.8 |
| 5 | *Bm*-RAL-2 (150 dpi) | 10.0 ± 5.0 | 2.3 ± 3.1 | 3.8 ± 3.9* | 3.4 ± 3.0 |
|  | *Bm*-103 (150 dpi) |  |  | 7.2 ± 5.2 | 2.3 ± 3.4 |
| 6 | Concurrent (90 dpi) | 4.8 ± 3.8 | 7.6 ± 6.2 | 6.5 ± 7.3 | 0.8 ± 1.0* |
| 7 | Fusion (90 dpi) | 6.1 ± 5.2 | 3.2 ± 3.9 | 1.6 ± 2.1 | 2.8 ± 3.4 |
| 8 | Concurrent (90 dpi) | 7.9 ± 6.3 | 5.7 ± 8.7 | 4.4 ± 3.6 | 1.5 ± 2.0 |
|  | Fusion (90 dpi) |  |  | 4.2 ± 3.1 | 2.7 ± 3.2 |
| 9 | Concurrent (150 dpi) | 12.4 ± 9.2 | 5.4 ± 5.3 | 6.1 ± 3.8 | 4.3 ± 6.5 |
|  | Fusion (150 dpi) |  |  | 2.2 ± 2.8*** | 4.9 ± 4.6 |

Asterisk/s denotes a statistically significant difference between the tested vaccine group in comparison to the alum control group and in the specific location (Lymphatics or Heart & Lungs) Mann–Whitney U Test, GraphPad Prism 6 (*, P ≤ 0.05; ***, P ≤ 0.001).
